# Non-contact physiological monitoring of preterm infants in the Neonatal Intensive Care Unit

**DOI:** 10.1038/s41746-019-0199-5

**Published:** 2019-12-12

**Authors:** Mauricio Villarroel, Sitthichok Chaichulee, João Jorge, Sara Davis, Gabrielle Green, Carlos Arteta, Andrew Zisserman, Kenny McCormick, Peter Watkinson, Lionel Tarassenko

**Affiliations:** 1 Institute of Biomedical Engineering, Department of Engineering Science, University of Oxford, Oxford, UK; 2 Neonatal Unit, John Radcliffe Hospital, Oxford University Hospitals Trust, Oxford, UK; 3 Visual Geometry Group, Department of Engineering Science, University of Oxford, Oxford, UK; 4 Nuffield Department of Clinical Neurosciences, University of Oxford, Oxford, UK

**Keywords:** Translational research, Medical imaging, Translational research, Medical imaging, Translational research

## Abstract

The implementation of video-based non-contact technologies to monitor the vital signs of preterm infants in the hospital presents several challenges, such as the detection of the presence or the absence of a patient in the video frame, robustness to changes in lighting conditions, automated identification of suitable time periods and regions of interest from which vital signs can be estimated. We carried out a clinical study to evaluate the accuracy and the proportion of time that heart rate and respiratory rate can be estimated from preterm infants using only a video camera in a clinical environment, without interfering with regular patient care. A total of 426.6 h of video and reference vital signs were recorded for 90 sessions from 30 preterm infants in the Neonatal Intensive Care Unit (NICU) of the John Radcliffe Hospital in Oxford. Each preterm infant was recorded under regular ambient light during daytime for up to four consecutive days. We developed multi-task deep learning algorithms to automatically segment skin areas and to estimate vital signs only when the infant was present in the field of view of the video camera and no clinical interventions were undertaken. We propose signal quality assessment algorithms for both heart rate and respiratory rate to discriminate between clinically acceptable and noisy signals. The mean absolute error between the reference and camera-derived heart rates was 2.3 beats/min for over 76% of the time for which the reference and camera data were valid. The mean absolute error between the reference and camera-derived respiratory rate was 3.5 breaths/min for over 82% of the time. Accurate estimates of heart rate and respiratory rate could be derived for at least 90% of the time, if gaps of up to 30 seconds with no estimates were allowed.

## Introduction

The World Health Organization defines term pregnancy as a delivery between 37 and 42 weeks of gestation.^1^ Gestational age is often computed as the number of completed weeks of pregnancy measured from the first day of the mother’s last menstrual period.^2,3^ Preterm birth, the primary focus of this paper, is defined as any birth prior to 37 weeks of gestation. Because the physiology and outcomes of preterm infants vary broadly, preterm birth is often subdivided as: late preterm, infants born between 34 and 37 weeks of gestation; moderate preterm, between 32 and 34 weeks; very preterm, between 28 and 32 weeks; and extremely preterm, infants born less than 28 weeks of gestation.^4^

Preterm birth is a major global health problem. It is estimated that more than one in ten of the world’s infants are born prematurely.^5^ It is the second leading cause of death in children under five years old^6^ and is the single most important cause of death in the first month of life.^4^ Preterm infants, especially those who are born very early, are often associated with motor and learning disabilities or visual and hearing impairment, accounting for approximately half of the disabilities in children and young adults.^4^ Preterm infants are often admitted into the Neonatal Intensive Care Unit (NICU) immediately after birth since they are not fully developed and tend to have medical conditions that require specialist care.^7^ Approximately one in seven babies born in England, Scotland and Wales in 2017 required specialist neonatal care.^8^ During the past decade, the number of admissions has continued to rise by approximately 13% each year.^8^ Constant nursing and medical supervision are provided to the infants until they are strong enough and ready to be discharged from the hospital. According to the last Neonatal Data Analysis Unit Report in the United Kingdom,^9^ the median hospital length of stay for extremely preterm infants is 93 days, 44 days for very preterm infants and 13 days for moderate and late preterm infants.

Patients in the NICU are unstable and have fluctuating vital signs. To monitor their physiological status, specialised medical equipment is used depending on their unique needs.^10^ The standard vital signs monitored usually include heart rate (HR), respiratory rate (RR), blood pressure, temperature and peripheral oxygen saturation ($Sp{O}_{2}$). A very low or high heart rate can indicate an underlying condition such as infection, pain or illness. Abnormal respiratory rate values are often associated with hypoxaemia (low level of oxygen in the blood), hypercapnia (high level of carbon dioxide in the blood) or acidosis (high level of acidity in the blood).^11^

**Fig. 1:**
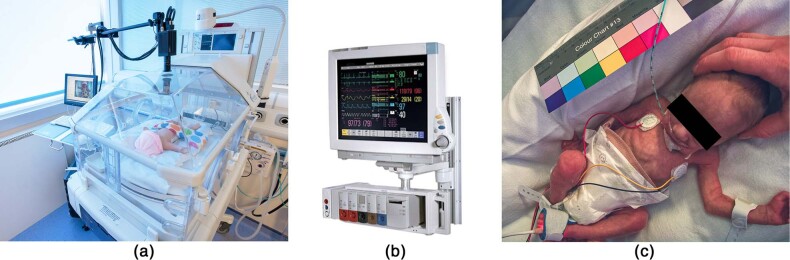
Data acquisition setup for a typical recording session in our clinical study. **a** A video camera was positioned over a specifically-drilled hole in the top surface of the study incubator. **b** Representative monitor used as a reference device to validate estimates computed from the camera data. **c** Sample image recorded from the video camera showing the ECG electrodes attached to the chest and a pulse oximeter attached to the patient’s left foot. Consent was obtained from the parents to use these images.

Continuous estimates of vital signs are typically provided by standard monitoring equipment (see Fig. 1b). Heart rate is usually computed from the Electrocardiogram (ECG). A pulse oximeter is often attached to the patient’s ear, finger or toe (see Fig. 1c) from which a Photoplethysmogram (PPG) signal is recorded and estimates of heart rate and $Sp{O}_{2}$ are computed. Respiratory rate is often computed from the Impedance Pneumography (IP) waveform, obtained by measuring changes in the electrical impedance of the patient’s thorax using the ECG electrodes. Clinical staff also make manual measurements every 4 h or up to every hour depending of the severity of the patient’s condition.

Conventional vital-sign monitoring technologies require the attachment of adhesive electrodes or transducers to the skin surface. The skin of preterm infants is fragile and very sensitive, especially for those born before 29 weeks of gestation when the bond between the attached sensor and dermis could be stronger than that between the dermis and epidermis.^12^ The attachment of sensors may damage the skin and increase the risk of developing an infection.^13^ Several technologies have been proposed for the non-contact monitoring of vital signs from the neonatal population, including methods based on capacitive-coupled electrodes, radar, laser, thermography and the use of off-the-shelf video cameras; a detailed summary can be found at.^14–16^

ECG monitoring using capacitive-coupled electrodes, first introduced in the 1960s^17^ and 1970s,^18^ does not require direct contact with the body and can enable the long-term monitoring of cardiac activity. Although attempts to increase the distance from the ECG electrodes to the subject being monitored have been proposed, most of the research in non-contact ECG for the neonatal population places the electrodes a few millimetres from the infants. The electrodes are usually embedded into the neonatal cot mattress,^19^ fabricated as a conductive fabric placed on top of the mattress,^20^ threaded into clothing or into the fabric covering the infant.^21^ Capacitive sensing is highly susceptible to body motion as poor sensor coupling can greatly change the capacitance and therefore negatively affect the recording of the ECG and the estimation of heart rate.^15,21^

Two main types of radar systems have been proposed for the recording of vital signs in the neonatal population: constant-frequency continuous-wave (CW) and ultra-wideband (UWB). In a typical CW radar system, a signal of known constant frequency is continuously transmitted in the direction of the infant’s chest. The transmitter is usually placed in front of the individual. As the chest moves away or towards the transmitter during inspiration and expiration, respectively, the reflected radar signal changes frequency as a result of the Doppler effect. The resulting signal modulated by the chest’s periodic motion can be used to estimate respiratory rate. Heart rate can also be estimated by analysing smaller chest wall movements due to the changes in position and volume of the heart during a cardiac cycle. UWB pulsed radar systems operate by generating a sequence of short pulses of finite duration, typically a few nanoseconds. Radar technology has several advantages, including the ability to penetrate through different materials such as clothing or through obstacles such as walls and mattresses, and it is not affected by ambient light levels.^22^

Radar systems can be located typically within metres of the subject.^23^ However, in a hospital environment, most of the reported research places the radar devices only a few centimetres away from the infant’s chest, typically attached to a tripod by the bedside^24^ or on top of the bed.^25,26^ Since radar systems estimate motion, it can be more difficult to measure the vital signs accurately in the neonatal population than in adults,^27^ as infants typically present more episodes of rapid movement. In practice, motion artefacts from the physical movement of the subject can result in interference in the radar’s output signal and may even be at the same frequency as the heart rate or respiratory rate.^28,29^

Chest wall movements induced by the pumping action of the heart, or by lung inflation when breathing, can also be measured with a Laser Doppler Vibrometer (LDV). By directing a laser beam onto a surface of interest, an LDV system can measure the vibration amplitude and frequency due to the motion of the surface.^30^ LDV prototypes have been used for the estimation of respiratory rate and heart rate in newborn infants.^31,32^ Recent developments can enable LDV systems to estimate heart rate, respiratory motion and gross physical activity, even in the presence of clothing.^33^ However, further research is needed to improve the accuracy, reduce the complexity, size and cost of LDV systems so that they can be implemented in a clinical environment.^15^

A thermal imaging camera measures the radiation emitted by objects in the long-infrared range of the electromagnetic spectrum ($8-14\;\mu$m). Since the amount of radiation emitted by an object increases with temperature, thermography can estimate the distribution and changes in temperature across the whole body.^34^ In the NICU, the estimation of respiratory rate is typically based on the analysis of the small temperature variations around the nose associated with the inspiration and expiration phases.^35,36^ Thermography has also been used to monitor the surface temperature of neonates^37^ and to study the evolution of necrotising enterocolitis (a condition in which tissues in the intestine become inflamed and start to die) and core temperature in premature infants.^38^ To ensure measurement accuracy and to reduce sensor-to-sensor variation, thermal imaging devices require calibration against temperature-controlled reference sources or industrial black body systems.^39,40^

With the cost of off-the-shelf digital video cameras continuing to decrease as the technology becomes more ubiquitous, research in non-contact vital-sign monitoring using digital camera sensors in the visible and near-infrared spectrum ($400\;-\;1000$ nm) has greatly expanded in recent years. It has been shown in the adult population that heart rate can be measured by the analysis of subtle colour and volume changes on the skin surface recorded by a video camera.^41–43^ Respiratory rate can be measured by the analysis of the movement of the torso.^43–45^ Peripheral arterial oxygen saturation has also been reported to be derived from signals obtained from a video camera at different wavelengths.^46,47^

Several studies have investigated the monitoring of vital signs of infants in a clinical environment. Heart rate was computed from 7 preterm infants for 30 seconds using a webcam and ambient light.^48^ A pilot study was carried out to investigate the estimation of heart rate from 19 preterm infants during short and stable periods between 1 and 5 min.^49^ Heart rate and respiratory rate estimation was previously reported for nearly 40 h of video recorded from two preterm infants during daytime under ambient light in the NICU.^50^ Other short studies have also been reported in the literature.^35,51–55^

The use of non-contact monitoring technologies for monitoring preterm infants can provide advantages over conventional vital-sign monitoring techniques. They can be integrated into a patient ward or a telemedicine system. In addition, they could be expanded to provide other bedside assessments such the infant’s physical activity, distress or pain. However, most of the research in video-based non-contact vital-sign monitoring has so far been performed over short-time periods (typically up to 5 min per recording) and under tightly controlled conditions with relatively still and healthy subjects. There are many challenges that remain before the technology can be deployed into clinical practice.^29^ A summary of video-based non-contact vital-sign monitoring methods can be found in refs. ^14,56,57^

We carried out a clinical study to evaluate the accuracy and the proportion of time that heart rate and respiratory rate can be estimated from preterm infants using only a video camera in a clinical environment, without interfering with regular patient care. The study consisted of the recording of 90 video sessions from 30 preterm infants, comprising a total recording time of approximately 426.6 h. It was carried out in the high-dependency area of the NICU at the John Radcliffe Hospital in Oxford (see Fig. 1). Each preterm infant was recorded under regular ambient light during daytime for up to four consecutive days. Since preterm infants are physiologically unstable, their vital-sign values can vary substantially in a short time period.

## Results

**Fig. 2:**
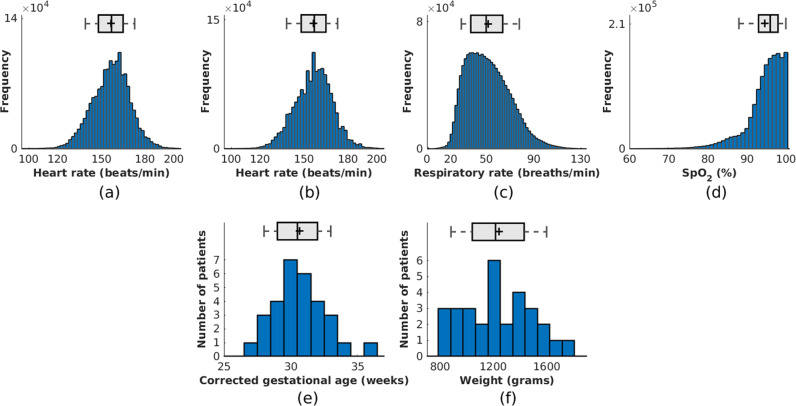
Distributions of vital signs for our clinical study. Above the histograms, a box plot bounds the 25% and 75% quartiles with whiskers marking 9% and 91% quantiles. The middle lines indicate the median whereas a plus mark indicates the mean. **a** Heart rate from ECG, **b** heart rate from PPG, **c** respiratory rate from IP and **d** oxygen saturation from the pulse oximeter. Distribution of **e** corrected gestational age and **f** infant weight collected on the first day of recording.

The clinical study ran for 15 months from February 2014 to May 2015. Table 1 provides a summary of the demographics of the patient population. A total of 90 sessions were recorded from 30 preterm infants. 18 out of 30 participants were male (60%). 18 out of 30 infants were White British (60%). Fig. 2e, f show the distribution of the corrected gestational age and of the weight of the participants, collected during the first day of recording. The corrected gestational age was computed by adding the number of weeks since birth on the first day of video recording to the gestational age at delivery. The range of corrected gestational age was 27.6–36.4 weeks, with a mean of 30.7 weeks. The weight of the infants varied between 830 and 1746 grams, with a mean of 1240 grams. Figure 2a–d show the distributions of vital-sign values (heart rate, respiratory rate and $Sp{O}_{2}$) recorded from the Philips patient monitor at 1 Hz over the entire clinical study.

**Table 1:** Summary of the population in the clinical study.

Description	Value
Total number of patients	30
Total recording sessions	90
Total video length (hours)	426.6
Average recording time per session (hours)^b^	4.9 ($\pm1.6$)
Average recording time per patient (hours)^b^	14.9 ($\pm5.7$)
Corrected gestational age (weeks)^b,c^	30.7 ($\pm2.0$)
Gender^a^	
Males	18 (60.0%)
Females	12 (40.0%)
Weight (grams)^c^	1240 ($\pm252$)
Ethnicity^a^	
White British	18 (60.0%)
White—any other background	2 (6.7%)
Asian or Asian British	2 (6.7%)
Black British or Black African	1 (3.3%)
Mixed—White and Asian	2 (6.7%)
Mixed—White and Black Caribbean	2 (6.7%)
Mixed—White British and Japanese	1 (3.3%)
Any other mixed background	2 (6.7%)

^a^N (percentage from total number of patients)

^b^mean ($$\pm$$std)

^c^On the first day of recording

The algorithms were developed and validated on half the study participants (the training set) and then tested on the other half (the test set). Table 2 gives a summary of the participant demographics for the training and test sets. The video recording information (date, time and duration) and patient demographics (ethnicity, corrected gestational age and weight) were chosen to be as balanced as possible between the two datasets. The training set was used to develop algorithms for pre-processing the video data and to optimise the global parameters of the vital-sign estimation algorithms.

**Table 2:** Summary of population demographics in the training and test sets.

Set	Number of subjects	Number of sessions	Total time (hours)^a^	Gender		Ethnicity^b^					
				Male	Female	W	B	A	WB	WA	O
Training	15	43	216.6	8	7	10	1	1	1	1	1
Test	15	47	210.0	10	5	10	$-$	1	1	2	1
Total	30	90	426.6	18	12	20	1	2	2	3	2

^a^Period during which both reference data and camera data were recorded simultaneously

^b^*W* White, *B* Black, *A* Asian, *WB* Mixed White & Black, *WA* Mixed White & Asian and *O* Other

**Fig. 3:**
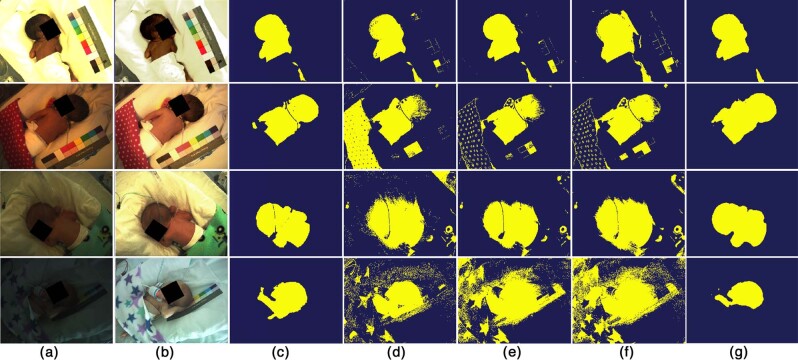
Comparison of skin segmentation algorithms under different lighting conditions. From top to bottom: a bright summer morning, an afternoon during autumn, a winter morning, and a dark winter afternoon. **a** Original images, **b** images with brightness increased manually so they can be displayed in this publication, **c** ground-truth segmentation. Results for the skin classifiers: **d** Naive Bayes, **e** Random Forests, **f** Gaussian Mixture Models, and **g** the proposed multi-task CNN. The baseline skin classifiers did not perform well in low-light scenarios, over-segmented the skin and generated false positives whose colours were similar to skin. The proposed multi-task CNN model produced more accurate skin labels. Consent was obtained from the parents to use these images.

Using our proposed multi-task Convolutional Neural Network (CNN), time periods during which the infant was present or absent from the incubator were automatically detected from the video recordings. Regions of interest (ROI) corresponding to skin were segmented from each video frame. These ROIs were used to extract cardiac-synchronous Photoplethysmographic Imaging (PPGi) and respiratory signals, from which heart rate and respiratory rate were estimated. Sample results for images recorded under varying lighting conditions are shown in Fig. 3. The results are shown together with the outputs from other commonly-used colour-based skin filters.^58,59^ Three classifiers were compared: Naive Bayes,^60^ Random Forests^61^ and Gaussian Mixture Models (GMMs).^62^

The three baseline skin filters classify each pixel as a skin pixel based solely on skin colours and provide a skin probability map, which can be thresholded to a binary label. Using two-fold cross validation on the data from 15 preterm infants included in the training set (see Table 2), our proposed multi-task CNN model achieved an accuracy of 98.8% for patient detection with an area under the receiving operating curve (AUC) score of 98.2%. For skin segmentation, the network yielded an average intersection-over-union (IOU) score of 88.6% and a pixel accuracy of 98.1%. Compared to the baseline colour-based skin filters, the proposed multi-task CNN model achieved a 3.1% higher pixel accuracy and a 12.7% higher IOU score. The performance of the different methods for patient detection and skin segmentation can be found in the supplementary information 1 provided for this paper.

Our CNN network was extended to detect time periods of clinical interventions and exclude them from the estimation process. Action recognition in video has been widely studied in the literature. A baseline method was developed based on the two-stream convolutional architecture for action recognition proposed by Simonyan and Zisserman^63^ and implemented using the VGG-M-2048 model.^64^ This method yielded an accuracy of 92.4% on our clinical study data. To identify the occurrence of clinical interventions, our proposed model fused information processed by the patient detection and skin segmentation network together with temporal information extracted from multiple-frame optical flow. Different sliding-window configurations and fusion strategies for the optical flow network were investigated. The configuration of a 5-second sliding window with 1-second step size and a temporal context fusion method yielded the highest performance with an accuracy of 94.5%. Detailed analysis of the performance of the different methods can be found in the supplementary information 2 provided for this paper.

The signal process algorithms to estimate heart rate and respiratory rate proposed in this paper, were developed on the data from half the participants (the training set) and evaluated on the remaining half (the test set), see Table 2. The protocol also required that video recording should not affect regular patient care, with priority given to the work of the clinical staff. Furthermore, the vital signs computed from the camera data could only be compared if the heart rate and respiratory rate values recorded by the reference monitoring equipment were consistent with each other. For example, differences between the heart rate computed from ECG and PPG can make the comparison with the camera estimates invalid. Therefore, “valid camera data” was defined as time periods for which the baby was present in the incubator, no clinical interventions were being carried out and the reference values for heart rate and respiratory rate derived from different monitoring equipment were in close agreement with each other (as described in refs. ^65,66^).

The original data consisted of 426.6 h of video recorded from 90 sessions. Ten recordings were discarded from the estimation process due to: reference data not recorded because of equipment malfunction (one session), patients undergoing blue-light phototherapy treatment (five sessions), and video out of focus due to video camera not properly calibrated at the start of recording (four sessions). Therefore, the resulting analysis was performed on only 80 sessions, corresponding to a total recording time of 384.3 h. The data were split into 192 h for the training set and 192.3 h for the test set.

**Fig. 4:**
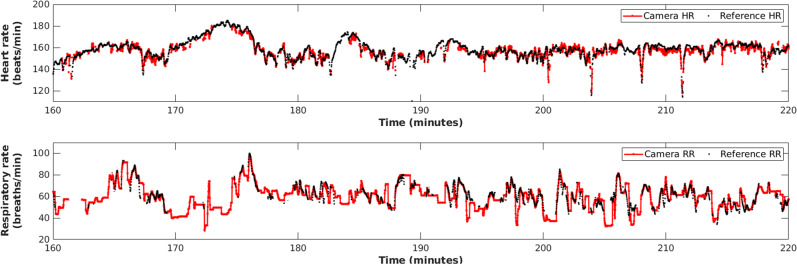
Vital-sign estimation for a sample 1-h recording from a 29-week female preterm infant with a weight of 1024 g on the first day of recording. For most of the time, good agreement is found between the reference signal and the camera-derived estimates. Episodes of short-term fluctuations are often associated with rapid infant movement.

Figure 4 shows the camera-derived heart rate and respiratory rate estimates compared with their corresponding ground-truth values for a 1-h sample period. The vital-sign estimates were computed from a video recorded from a female preterm infant of 29-week gestation and 1024 g weight on the first day of recording. For most of the segment, a good agreement is found between the reference signals and the camera-derived estimates. Episodes of short-term fluctuations are often associated with rapid infant movement, spontaneous movement patterns such as body stretching or other motion-related artefacts.^67,68^ The respiratory rate estimates varied between 20 and 100 breaths/min and generally agree with the reference respiratory rate.

**Fig. 5:**
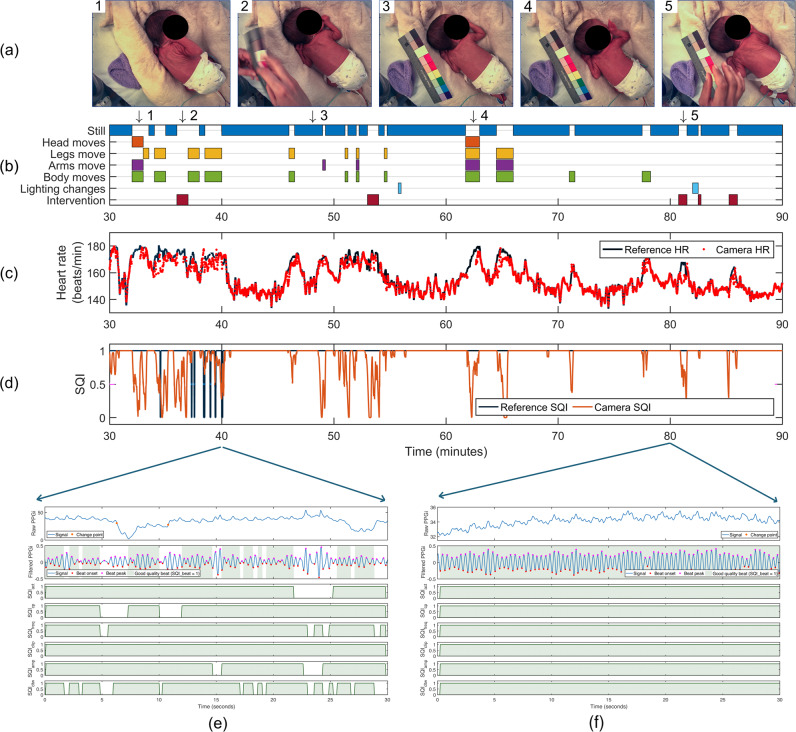
Heart rate estimation for a sample 60-min period from a male preterm infant with a gestational age of 28 weeks and weight of 1220 g. **a** Video frames corresponding to the time in the plot below. **b** Timeline of typical patient and clinical staff activity, manually annotated minute-by-minute by the authors. **c** Comparison of the reference heart rate and the camera-derived heart rate estimates computed using the AR best model. **d** Signal quality assessment for the heart rate estimates for the entire period of 60 min. Detail of the signal quality assessment for two 30-second PPGi windows taken from **e** a period during which the infant was active, and **f** a quiet period during sleep. Consent was obtained from the parents to use these images.

Figure 5 shows the process of computing heart rate using the Autoregressive (AR) best model for a 60-min video recorded from a male preterm infant of 28-week gestation and 1220g weight. Manual minute-by-minute annotation of the typical patient and clinical staff activity are presented, including periods of infant motion, clinical interventions and changes in the ambient light. The figure also shows the detail of the quality assessment for two 30-second PPGi windows, taken from periods during which the infant was active (Fig. 5e) and was quietly sleeping (Fig. 5f). During periods of patient movement, the quality of the PPGi signal was automatically identified as poor and, therefore, a reliable heart rate estimate could not be computed. In contrast, during period for which the infant was less active, reliable heart rate estimates with high accuracy could be computed.

**Fig. 6:**
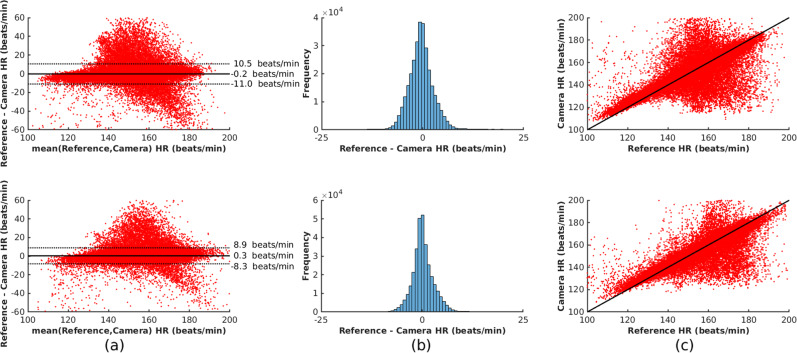
Comparison between the reference and camera-derived heart rate estimates using the AR best model method for the training set (top row) and test set (bottom row). The **a** Bland-Altman plot, **b** Histogram of the differences between the two heart rate estimates and **c** Correlation plot show minimal bias and a positive correlation between the two measurements.

**Fig. 7:**
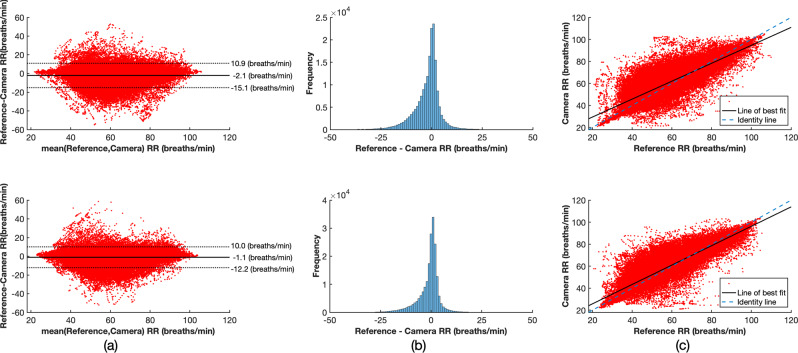
Comparison between the reference and camera-derived respiratory rate for the training set (top row) and test set (bottom row). The **a** Bland-Altman plot, **b** Histogram of the differences between the two respiratory rate estimates and **c** Correlation plot show minimal bias and a positive correlation between the two measurements.

Figure 6 compares the reference and camera-derived heart rate using an algorithm based on AR best model. The mean difference between the two measurements was 0.2 and 0.3 beats/min for the training and test sets, respectively. A positive correlation was found with a correlation coefficient of 0.86 for the training set and 0.93 for the test set. Similarly, Fig. 7 shows the respiratory rate estimation comparison. The estimated values were distributed across the expected physiological range for the neonatal population, taken to be from 18 to 120 breaths/min. A positive correlation was also found with a correlation coefficient of 0.85 and 0.89 for the training and test sets, respectively.

Table 3 summarises the results for all the vital-sign estimation algorithms. For the process of heart rate estimation, 72.9% (139.9 h) of the total recording time (192.0 h) was considered valid for the training set. For the test set, 63.6% (122.3 h) of the total recording time (192.3 h) was considered valid. The AR best model method slightly outperformed all the other methods, with a mean absolute error (MAE) of 2.9 beats/min and 2.3 beats/min for the training and test sets respectively and mean absolute deviation (MAD) of 2.9 beats/min and 2.3 beats/min for the same datasets. The AR best model method has a strict set of rules that discard noisy time periods, hence its high accuracy comes at a cost of a lower proportion of estimated time. For the rest of the methods, MAE varied between 2.6 beats/min and 4.7 beats/min. Heart rate was estimated for up to 69.1% and 79.4% of the total time the video data were judged as valid in the training and test set, respectively. In contrast, the poor quality of the reference respiratory rate, as computed by the monitoring equipment, severely restricted the time during which the process of estimating respiratory rate from the video camera could be evaluated. Only 37.1% (71.2 h) of the total recording time (192.0 h) was considered valid for the training set. Similarly, 34.6% (66.4 h) of the total recording time (192.3 h) was considered valid for the test set. The MAE ranged from 4.5 breaths/min to 3.5 breaths/min for the training and test sets, respectively.

**Table 3:** Summary of the vital-sign estimation results for all recording sessions.

Vital sign	Dataset or algorithm	Total recording time (h)	Image and signal pre-processing (h, %)^a^			Vital-sign estimation (h, %)		Error^c^	
			Poor reference	Subject absence	Clinical intervention	Valid camera data^a^	Estimated time^b^	MAE	MAD
Heart rate	Training set	192.0 h	14.3 h, 7.4%	16.3 h, 8.5%	21.5 h, 11.2%	139.9 h, 72.9%			
	Beat counting	"	"	"	"	"	96.7 h, 69.1%	4.1	4.5
	FFT	"	"	"	"	"	96.7 h, 69.1%	3.4	3.8
	AR dominant pole	"	"	"	"	"	96.7 h, 69.1%	4.7	4.8
	AR best model	"	"	"	"	"	87.8 h, 62.8%	2.9	2.9
	Test set	192.3 h	20.1 h, 10.4%	27.9 h, 14.5%	22.0 h, 11.5%	122.3 h, 63.6%			
	Beat counting	"	"	"	"	"	97.1 h, 79.4%	3.3	3.8
	FFT	"	"	"	"	"	97.1 h, 79.4%	2.6	2.8
	AR dominant pole	"	"	"	"	"	97.1 h, 79.4%	4.0	4.2
	AR best model	"	"	"	"	"	92.2 h, 75.4%	2.3	2.3
Respiratory rate	Training set	192.0 h	106.6 h, 55.6%	16.3 h, 8.5%	21.5 h, 11.2%	71.2 h, 37.1%	51.4 h, 72.2%	4.5	3.8
	Test set	192.3 h	104.3 h, 54.3%	27.9 h, 14.5%	22.0 h, 11.5%	66.4 h, 34.6%	54.8 h, 82.5%	3.5	3.0

^a^Percentage with respect to the total recording time

^b^Percentage with respect to the valid camera data

^c^beats/min for HR and breaths/min for RR

Table 4 presents the vital-sign estimation results according to the ethnicity of the patients recruited to the study. Compared with the test set, the training set had a slightly wider range of ethnic groups. Subjects in the non-White groups in the test set had lighter skin tone than those in the training set. Generally, the errors in heart rate estimation for patients with lighter skin tone were lower than for the other ethnic groups.

**Table 4:** Vital-sign estimation results for all the recording sessions according to ethnicity.

Ethnicity	Number of subjects	Number of sessions	Heart rate			Respiratory rate		
			Error (beats/min)		Estimated time (%)	Error (breaths/min)		Estimated time (%)
			MAE	MAD		MAE	MAD	
Training set								
White	9	25	2.5	1.9	65.3%	4.7	3.8	75.1%
Asian	1	1	3.3	1.8	73.4%	5.7	5.5	71.2%
Black	1	3	3.8	2.8	56.4%	4.1	3.5	59.6%
Mixed White & Asian	1	4	3.7	3.0	63.9%	4.2	3.8	67.3%
Mixed White & Black	1	2	5.6	5.2	48.6%	4.9	4.0	47.4%
Mixed Others	1	3	3.2	2.7	55.3%	3.8	3.2	61.7%
Test set								
White	10	30	2.3	1.8	74.6%	3.5	3.0	85.2%
Mixed White & Asian	2	3	2.2	1.8	79.1%	3.9	3.6	80.3%
Mixed White & Black	1	5	3.3	2.4	60.8%	3.2	2.5	78.1%
Mixed Others	1	4	1.7	1.3	87.7%	3.3	2.9	81.3%

**Fig. 8:**
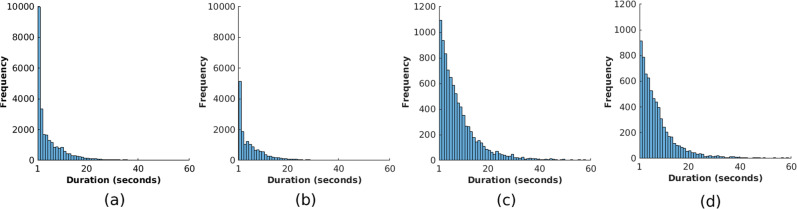
Gaps in time for the estimation of vital signs. Gaps in the heart rate estimates for **a** the training set and **b** the test set during valid time periods. Gaps in the respiratory rate for **c** the training set and **d** the test.

Figure 8 shows the histogram of the periods for which heart rate and respiratory rate could not be estimated. Most of the gaps were less than 30 seconds. If gaps of up to 30 seconds were allowed, the percentage of the estimated time with respect to the valid time increased from 62.8% to 90.0% for the training set and from 75.4% to 96.9% for the test set for heart rate. For respiratory rate, it increased from 72.2% to 94.5% for the training set and from 82.5% to 96.5% for the test set.

## Discussion

This paper proposes non-contact algorithms for estimating heart rate and respiratory rate from preterm infants in an unconstrained and challenging hospital environment. The process involved the extraction of cardiac and respiratory signals from the video camera data via deep learning algorithms and the development of robust techniques for the estimation of the vital signs. The proposed multi-task deep learning algorithms performed three tasks that provided essential information for the automatic extraction of vital signs from a video camera in a hospital environment: the detection of the patient in the video frame, the automated segmentation of skin areas and the detection of time periods during which clinical interventions were performed by the attending hospital staff. Two open-source custom software packages were developed:^69^ the first is a custom-built semi-automatic code for labelling the skin regions of people in images; and the second software code is for annotating the time periods during which clinical interventions were present in videos.

The automatic detection of a patient in the video frame and the accurate segmentation of skin regions are essential requirements for the successful estimation of vital signs in a hospital environment. The proposed multi-task CNN was able to locate the infant and identify suitable time periods of vital-sign estimation. It demonstrated robustness in tracking pose variations and discarding areas in the image frame that corresponded to other individuals such as parents or clinical staff. One potential disadvantage of our model is the difficulty of segmenting small skin regions as the CNN architecture successively down-samples feature maps in the network. By learning individual visual cues, the proposed CNN can be expanded to recognise each individual preterm infant and support the simultaneous estimation of vital signs from multiple patients. This can be integrated into the hospital information system or a telemedicine infrastructure, lowering further the costs of implementation in a clinical environment. Automatic patient recognition can be more challenging to implement in other non-contact monitoring technologies based on laser or radar where the location of the target to monitor can be an issue.^27^

The proposed system was robust to the typical daytime changes in lighting conditions of the hospital ward. Figure 3a shows some examples of the original recorded images under different levels of illumination, from a bright summer morning to a dark winter late afternoon. Even under low-light conditions, the proposed CNN was able to detect the patient, segment the skin areas and compute the vital-sign estimates. In comparison, the reference baseline algorithms performed poorly in low-light conditions as colours were distorted and the difference between skin and non-skin pixels became less distinguishable. The proposed CNN network did not produce noisy or grainy skin labels as it processed the whole image at once. Although the proposed system was robust under low-light ambient conditions, further research is needed to validate its accuracy under completely dark ambient conditions such as during the night. The system can use a video camera with an imaging sensor sensitive to the near-infrared spectrum and a matching infrared external illumination source that is not visible to the human eye (above $800$ nm). Indeed this is the approach used in the commercial version of our system.^70^

Due to high melanin concentration, dark-colour skin absorbs more energy, therefore less energy is reflected back from the skin surface. This leads to a low signal-to-noise ratio for the signals recorded from an individual with dark skin colour using optical-based technologies such as video cameras. Although our proposed system was robust to the different ethnicities of the patients in our study, the population was comprised mostly of light-skin preterm infants. Further research is needed to validate the algorithms on dark-skin subjects. One advantage of radar-based systems is that they are not affected by the colour of the subject’s skin.

The multi-task CNN model exhibited similar performance to the CNN models trained individually for a single task. The joint network achieved an improvement of 1.7% in accuracy and 0.3% in AUC score for patient detection and an improvement of 0.9% in IOU score for skin segmentation compared to that of the single-task networks. Similar results were observed in the multi-task network of Gkioxari et al.^71^ As expected, the joint network did not show a bias towards one individual task. The multi-task network performed both tasks twice as fast as a cascade of two single-task networks. Data augmentation was found to substantially improve the performance of the network as well as the quality of segmentation results. With data augmentation applied, 30.5% and 11.4% improvements in IOU were observed for the single-task model and the multi-task model, respectively. Without data augmentation, the CNN produced coarser segmentation results. This might be due to the small number of patients in our dataset, making it difficult for the network to learn the generic structure of the infants in the incubator.

By applying spatio-temporal fusion to the process of detecting clinical interventions, we expected the convolution layers of the patient detection and skin segmentation network to work as a generic feature extractor. Surprisingly, the performance of the spatio-temporal network was found to be lower than that of the optical flow network before fusion. In addition, fusing information from multiple frames performed worse than using just a single frame. It is possible that the high-level convolutional features were too specific to the original patient detection and skin segmentation tasks. They may not carry meaningful information for the detection of intervention periods. By stacking the feature maps of multiple video frames together, the network found it difficult to learn, possibly because of the large numbers of free parameters. Most false positives in the detection of clinical interventions (incorrectly-identified clinical interventions) were found among the following scenarios: infant very active or crying; position of the camera adjusted by clinical staff; abrupt change in lighting conditions when fluorescent lights were switched on or off, or window blinds were opened or closed. Daylight illumination could also change quickly when the sky went from clear to cloudy, and vice versa. Other sources of error occurred when clinical staff near the incubator cast shadows on the infant, or the incubator was disturbed when clinical staff came to check equipment or to manually record vital-sign values.

The changes in lighting conditions and the movement of the camera or incubator caused abrupt changes in optical flow. False negatives (clinical interventions missed) occurred in the following scenarios: parents holding the infant in their arms during their visits; clinical staff providing stimulation by touching the infant with their hands not moving inside the incubator for a short time; clinical staff’s hands not touching the baby during the intervention; nursing staff holding a timer during manual respiratory counting. The errors were likely to have been caused by small changes in optical flow during the above scenarios, such that the network misclassified an intervention event as a non-intervention. A summary of the typical daily nursing activities in the NICU can be found in the supplementary information 3 provided for this paper.

The use of the entire skin area allowed the estimation of vital signs in a challenging clinical environment such as the NICU. Heart rate and respiratory rate could be estimated with high accuracy during quiet and stable periods. As expected, the estimation of vital signs using a video camera was affected by several factors such as motion artefacts, ambient light changes, interventions by the clinical staff and other external factors. Random body movements or other motion artefacts not only affect most conventional vital-sign measurement methods (for example Impedance Pneumography and ECG),^29^ but also significantly affect all the other non-contact monitoring technologies.^14–16^ Most of the gaps for which vital signs could not be estimated by our proposed system were shorter than 30 seconds, as shown in Fig. 8. Our system provides the clinical staff with high-quality estimates with minimal interruption and trends of the patient’s physiology can be constructed for long periods of time. Overall, the errors presented in this paper are consistent with our previous results in the NICU population^50^ and with adults in dialysis.^43^

The complete multi-task CNN proposed in this paper runs in realtime at the same rate of 20 fps as the recording video camera on an Nvidia Titan 6 GB and at 60 fps on a Nvidia 1080Ti. Faster performance can be achieved if needed with dual GPUs or by reducing the size of the images recorded. The signal processing algorithms to estimate vital signals require an initial delay of 8 and 10 seconds for heart rate and respiratory rate, respectively. Following the initial delay, the estimates are computed on a second-by-second basis. The results presented in this paper were computed retrospectively using software developed using Matlab. However, a realtime implementation was developed in the C/C++ programming language to run the algorithms on desktop computers and mobile devices.

Most of the current work in non-contact vital-sign monitoring using video cameras has been performed over short-time periods (typically between 1 and 5 min per recording), under tightly controlled conditions with relatively still and healthy subjects. Most of the studies that analyse the neonatal population in a clinical environment record short videos from a small number of participants (typically under 10). We evaluated the accuracy and the proportion of time that heart rate and respiratory rate can be estimated from 30 preterm infants in the clinical environment of the NICU, without interfering with regular patient care. We recorded 90 videos sessions, comprising a total recording time of approximately 426.6 h, the videos correspond to only 30 preterm infants. Before video-based vital-sign monitoring is accepted and deployed in the clinical environment, studies with larger numbers of infants are needed, with a comprehensive range of ethnicity, gestational age, gender, skin colour and neonatal complications.

Camera-based technologies are ubiquitous, low-cost and capable of high performance. Non-contact monitoring using video cameras has the potential to be expanded to monitor an infant’s physical activity, distress, pain and to detect other adverse clinical events such as apnoea (pauses in breathing) or bradycardia (low heart rate). Apart from the monitoring of vital signs, a video camera could also be used to monitor an infant’s physical activity, distress and pain. Current procedures for pain assessment are based on the subjective observations of changes in vital signs, behavioural indicators and the infant’s state of arousal.^72,73^ To continuously monitor these and other medical conditions for 24 h each day, there is a need for imaging sensors (with external illumination). In addition to non-contact vital-sign monitoring, video camera technology and algorithms could be used to evaluate these parameters objectively.

## Methods

### Clinical study

Our clinical study was part of a research programme in the Oxford University Hospitals NHS (National Health Service) Foundation Trust and the Oxford Biomedical Research Centre (BRC). The research was compliant with the relevant government and regulations of the Oxford University NHS Foundation Trust. The study was approved by the South Central Oxford Research Ethics Committee under reference number 13/SC/0597.

#### Study design and protocol

The clinical study was designed to run without affecting regular patient care. Preterm infants who participated in the study were required to be nursed in a designated study incubator in the high-dependency area of the NICU at the John Radcliffe Hospital in Oxford. The aim of the study was to monitor 30 preterm infants for up to four consecutive days. The study protocol allowed the algorithms to be developed on half the participants (15 patients) and to be evaluated on the remaining half. The clinical team recruited the infants based on the British Association of Perinatal Medicine’s Categories of Care 2011.^74^ The participants were double-monitored with a digital video camera and the standard patient monitoring devices. The study was performed during daytime under regular ambient light conditions.

Participants needed to satisfy all of the following criteria: born less than 37 weeks of gestation; requiring high-dependency care; requiring continuous monitoring of heart rate, respiratory rate and oxygen saturation; requiring to be nursed naked. The study excluded any infants who presented life-threatening conditions that prevented the continuous monitoring in the high-dependency area of the NICU. Consent was required to be given by the babies’ parents prior to any recording. Parents whose infants fulfilled the inclusion criteria were approached by the study personnel (NICU clinicians) and given full verbal and written information about the study.

All the standard patient monitoring and care were continued throughout the study session. The setup of all the research equipment (video recording and data storage) was designed to minimise the inconvenience to clinical staff during the study. No additional sensors were attached to the infants. Access to the incubator was not in any way restricted by the position and location of the video camera and the associated equipment. Video recording could be temporarily paused, or the video camera could be temporarily covered, at the discretion of clinical staff, during some clinical procedures such as phototherapy (for treating jaundice—yellow appearance of the skin), intravenous (IV) cannulation or when the infants were taken out of the incubator for cuddling by their parents (kangaroo care). If the infants were to be transferred to another unit, video recording was terminated and data were recorded until that point.

#### Instrumentation

Preterm infants were cared for in a designated Giraffe OmniBed Carestation incubator (General Electric, Connecticut, USA). A modification was made to the incubator by drilling a small hole in the top plastic panel of the incubator’s canopy. This allowed a video camera to be positioned inside the incubator’s chamber in order to film the infants without reflection and attenuation from the perspex layer (see Fig. 1). The modification to the incubator was approved by the Medical Research Ethics Committee (MREC). After the modification was carried out, a series of humidity and temperature tests were performed over a period of 2 weeks to ensure that the incubator was safe for clinical use and had the same level of environmental control within the chamber as a standard unmodified incubator.

The data acquisition system (see Fig. 1) consisted of a trolley carrying the video camera and two recording workstations. One workstation was used to record video from the camera, and the other for recording reference vital signs from the patient monitor. A medical-grade keyboard and mouse (Accumed - Accuratus, UK) were used with both workstations. These devices complied with the IP67 standard for dust and water protection and the JIS Z 2801 test for antimicrobial activity of plastics.

Video recordings were acquired using a JAI 3-CCD AT-200CL digital video camera (JAI A/S, Denmark). The camera employs three Sony ICX274AL 31/1.8” image sensors (Sony, Japan) to measure the light intensity of each colour channel (red, green and blue) separately. The video camera was equipped with a VS Technology SV-0614H lens (VS Technology, Japan) which allowed full control of focal length and aperture. Before, and occasionally during video recording, the attending clinical staff were required to adjust these parameters to ensure that the infant was in focus and that the brightness level was adequate. The video camera system acquired 24-bit lossless colour images (8-bit per colour) at a resolution of 1620$\times$1236 pixels and at a rate of 20 frames per second. The video was recorded using a frame grabber board with a Field Programmable Gate Array (FPGA) integrated circuit (Xilinx, California, USA). The video recording software was designed and developed by the authors to work with the continuous transmission of large data streams without image corruption or data loss. A typical 1-h recording produced approximately 408 GB of data.

Reference vital signs (heart rate, respiratory rate and oxygen saturation) were recorded using a Philips IntelliVue MX800 patient monitor (Philips, Netherlands). The patient monitor was installed with a Philips IntelliVue Multi-Measurement Module to record ECG and IP signals, and a Masimo SET $Sp{O}_{2}$ Vuelink IntelliVue measurement module (Masimo, California, USA) to record PPG. The patient monitor was connected to a workstation via a serial interface. The ixTrend software (Ixellence GmbH, Germany) was used to record the data streams generated by the Philips patient monitor. The following waveforms were recorded: 1-lead ECG signal (at 500 Hz), IP signal (at 64 Hz) and the PPG signal (at 125 Hz). The following physiological parameters were recorded at a rate of 1 Hz: heart rate from ECG, heart rate from PPG, respiratory rate from IP and $Sp{O}_{2}$ from the pulse oximeter.

### Overview of the proposed framework

The proposed framework consists of two CNN models followed by signal processing methods to compute heart rate and respiratory rate estimates. The first multi-task CNN model analysed the input video to identify suitable time periods for which the location of the patient within the video frame could be detected and tracked. Areas of the video frame that contained skin were subsequently segmented for further analysis. The outputs of the first network were combined with a second CNN model with optical flow for identifying time periods during which clinical interventions occurred. These periods were discarded from the estimation process. Once the location of the patient was computed, and quiet periods in the video recordings were identified, several cardiac and respiratory signals were extracted from each video frame. Methods to assess the quality of cardiac and respiratory pulses are proposed so that periods of high activity or motion artefacts could be excluded from the vital-sign estimation process. Finally, heart rate and respiratory rate were estimated using data fusion algorithms by analysing several regions of interest (ROI) across the patient’s skin areas and along the upper torso.

The clinical study protocol allowed the algorithms to be developed only on half the participants. The proposed CNN networks for patient detection, skin segmentation and clinical intervention detection described below, were developed and evaluated with a two-fold cross-validation procedure using only the 15 preterm infants dataset labelled as “training” in Table 2. In contrast, the signal processing methods to estimate vital signs were developed using the 15 preterm infant dataset labelled as “training” and evaluated using the remaining 15 preterm infant dataset labelled as “test” as described in Table 2.

### Patient detection and skin segmentation

**Fig. 9:**
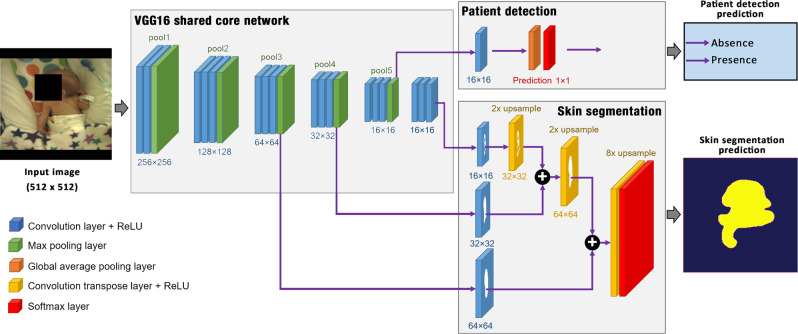
The proposed CNN model extended the VGG-16 network with two branches: skin segmentation branch, implemented using a fully convolutional network; and a classification branch, implemented using global average pooling over feature maps. The network was modified to evaluate the segmentation branch only if the classification branch found a preterm infant in the image. Consent was obtained from the parents to use these images.

The first proposed CNN network performed the joint task of image classification and segmentation. For each video frame, the network computed a decision on whether the infant was in the scene, together with the segmented skin regions if the infant was found. Our multi-task network has a shared core network, implemented using the VGG-16 architecture,^75^ with two output branches: the patient detection branch, implemented using global average pooling; and the skin segmentation branch, implemented using hierarchy upsampling of image features across the shared core network (see Fig. 9). The VGG-16 network was originally developed for image classification and was previously trained on 1.3 million images of the ImageNet dataset. It has been recognised as a generic feature extractor and has demonstrated good generalisation in transfer learning.^75^

Our extension to the VGG-16 network followed that of the fully convolutional network.^76^ Several modifications were needed to enable the skin segmentation branch to perform pixel-level segmentation on the output of the shared core network. All fully-connected layers in the VGG-16 network were converted into convolution layers by having them perform convolution operations on the input data. These layers then produced a spatial output map with the spatial coordinates preserved (see Fig. 9). The last convolution layer was modified to produce 2-class-scoring outputs for the skin and non - skin classes. Although a sufficiently large input image is typically required to achieve accurate segmentation results,^76^ the spatial resolution used by our network was limited by the amount of memory and computational power required during training. The input images were resized from their original resolution of $1620\;\times\;1236$ to $512\;\times\;512$ pixels. The original aspect ratio of the images was maintained by adding black pixels at the top and bottom of the image. The pixel-level skin segmentation output was resized back to the original image resolution, so that the vital-sign estimation algorithms could work on the original colour images.

The patient detection branch was implemented using global average pooling for classification, similarly to refs. ^77,78^ In our implementation, a $1\times 1$ convolution layer with two outputs was added on top of the pool5 layer (the layer before the original fully-connected layers in the shared core network). The $1\times 1$ convolution layer performed a linear combination across feature maps in order to reduce the size of the feature dimension. The $1\times 1$ convolution layer was followed by a global average pooling layer, which averaged out the spatial information, resulting in an output vector fed to a softmax layer. The patient detection branch therefore, produced a 2-output vector of class-scoring estimates related to the presence or the absence of the infant in the video frame.

The skin segmentation branch was implemented using a fully convolutional network for image segmentation which performed a series of spatial upsampling steps from cross-network feature maps.^76^ It employed convolutional transpose layers to project feature maps onto a larger-dimensional space and produce pixel-level labelling of skin regions. Our implementation followed that of Long et al.^76^ Given that the size of the input to the network was $512\;\times\;512$ pixels, the feature maps of the last convolutional layer in the shared core network had a spatial size of $16\;\times\;16$ pixels (a factor of 32 reduction of the input size). A $1\times 1$ convolution layer with 2 outputs was first added on top of this layer to produce a coarse prediction of non-skin and skin classes at a $16\;\times\;16$ pixels reduction. As the feature maps of the pool4 and pool3 layers in the shared core network had a spatial size of $32\times 32$ and $64\;\times\;64$ pixels, respectively, a $1\;\times\;1$ convolution layer with 2 outputs was added on top of each of these layers. This produced two additional predictions of skin and non-skin classes at finer resolutions of $32\;\times\;32$ and $64\;\times\;64$ pixels respectively.

The coarse prediction at $16\;\times\;16$ pixels was spatially upsampled through a convolutional transpose layer with a factor of 2, producing a finer prediction at $32\;\times\;32$ pixels. The resulting prediction was later fused with the prediction of the pool4 layer at $32\;\times\;32$ pixels. Subsequently, in the same manner, the prediction fused from these two layers, at $32\;\times\;32$ pixels, was spatially upsampled by a factor of 2, producing a finer prediction at $64\;\times\;64$ pixels. The resulting prediction was then fused with the prediction of the pool3 layer. This resulted in a prediction at a factor of 8 of the original resolution ($64\;\times\;64$ pixels). Finally, a convolutional transpose layer with a factor of 8 was added in order to obtain a final prediction at the same spatial size as the input image ($512\;\times\;512$ pixels). The network was completed by a softmax layer, which produces per-pixel class-scoring estimates. The skin segmentation branch was executed only if the presence of the infant was identified by the patient detection branch.

To generate ground-truth data for training the proposed network, a database was created consisting of positive images in which infants were present (with pixel-level skin labels) and negative images in which infants were absent. Three annotators were asked to label the video images. Due to the large amount of data, we developed a custom open-source semi-automatic annotation tool, available at ref. ^69^ To address the trade-off between annotation effort and sufficient variation in the input images, one video frame was extracted every 6 min corresponding to a total of 2269 images. The annotations of skin regions from the three annotators were combined to form the positive images. Images were regarded as positive if two or more annotators provided skin labels. With this criterion, 1718 out of the 2269 images (76%) were labelled as positive. For each image, a pixel was regarded as skin if at least two annotators agreed, otherwise the pixel was marked as non-skin. The inter-annotator agreement was 96.7%.

To create the dataset of negative images, we used the nurses’ notes to extract images during time periods for which the infants were taken out of the incubator. These periods included clinical activities such as kangaroo care, infant taken to another clinical study and video camera covered by the nurses. For the 15 infants in the training set, these periods accounted for approximately 23.5 h. Images were taken every 20 seconds, corresponding to a total of 4227 images. The same annotation strategy as in the previous step was used for the three annotators to classify all the images as infant or non-infant. The images for which two or more annotators agree were regarded as negative. With this scheme, 2885 negative images were selected. The inter-annotator agreement was 99.47%. There were several images where the ambient light in the NICU was very dimmed (even darker than Fig. 3a), therefore some annotators overlooked an infant in the scene. To create a balanced dataset, 1718 negative images were randomly selected from the pool of 2885 negative images. Therefore, the total dataset consisted of 3436 images (1718 positive images and 1718 negative images) and split equally between the training and test set.

Multiple variations of each training image were generated. We employed three data augmentation techniques during training: rotational, mirroring and lighting augmentation. The total number of the resulting dataset was 44,668 images. CNNs have several degrees of translation and rotation invariance as a result of the convolution and pooling processes, which progressively increase the level of abstraction of the image.^79^ In order to encourage the network to learn rotational invariance, seven additional images were generated for each original image by rotating the image at 45-degree increments between 0 and 360 degrees. In order to encourage the network to learn the symmetry of the human body, two additional images were generated by mirroring each original image with respect to the centre of the image on the *x*-axis and *y*-axis.

By varying the lighting characteristics in each image, the network could be made invariant to illumination changes from both natural and artificial light sources. The augmentation was performed by converting the original image into the Hue-Saturation-Lighting (HSL) colour space, scaling the lightness component and then converting the image back into the RGB colour space as in ref. ^80^ The average lightness component was calculated for each training image. A lightness range was defined by the minimum and maximum of the averages of the illuminant component computed across all the images in the training set. The range was divided into four uniform intervals and the mean of each interval was calculated. For each image, if the average lightness fell in one of the four intervals, three additional images were generated by scaling the lightness component in the HSL space using the values calculated from the three other intervals.

The CNN network was trained jointly using a unified multi-objective loss function composed from the two CNN models. Given an input image $x$, the output ${y}_{\det }$ = {${d}_{0},\,{d}_{1}$} of the patient detection branch is a two-class softmax probability vector. Suppose that $B$ = {${b}_{0},\,{b}_{1}$} is a ground-truth label where $B$ = {$1,\,0$} indicates the absence of the infant in the image and $B$ = {$0,\,1$} indicates the presence of the infant in the image. The loss function for the patient detection branch was defined as the multinomial logistic loss of the softmax output:^62^

1 $${{\rm{Loss}}}_{\det }=-{b}_{0}\,{\rm{log}}({d}_{0})-{b}_{1}\,{\rm{log}}({d}_{1})$$

Since the output of the skin segmentation branch was a pixel-level skin label whose spatial size was equal to the input image size, the loss was summed across all pixels. As the number of non-skin pixels was larger than that of skin pixels, the contribution to the loss of the skin class was then weighted according to the ratio of the number of ground-truth non-skin pixels, ${N}_{{\rm{non}}-{\rm skin}}$, to that of ground-truth skin pixels, ${N}_{{\rm{skin}}}$. Given that ${\mathscr{P}}$ is a set of pixels in the input image $x$, the output ${y}_{{\rm{seg}}}$ = {${s}_{0},\,{s}_{1}$} of the skin segmentation branch is a two-class softmax probability vector for each pixel, where the subscripts 0 and 1 denote the non-skin and skin classes respectively. $L=\{{l}_{0},\,{l}_{1}\}$ is the ground-truth skin annotation where ${l}_{0},\,{l}_{1}\in \{0,1\}$. The loss function of the skin segmentation branch was defined as:

2 $${{\rm{Loss}}}_{{\rm{seg}}}=-\sum _{i=1}^{{\mathscr{P}}}\,{l}_{0}(i)\,{\rm{log}}\,({s}_{0}(i))-\lambda \sum _{i=1}^{{\mathscr{P}}}\,{l}_{1}(i)\,{\rm{log}}\,({s}_{1}(i))$$

where the weighting factor $\lambda$ is defined as:

3 $$\lambda =\frac{{N}_{{\rm{non-skin}}}}{{N}_{{\rm{skin}}}}.$$

The unified multi-objective loss function was defined as the weighted sum of the two loss functions:

4 $${\rm{Loss}}(f(x),{G}_{x})={\alpha }_{\det }{{\rm{Loss}}}_{\det }({y}_{\det },{B}_{x})+{\alpha }_{{\rm{seg}}}{{\rm{Loss}}}_{{\rm{seg}}}({y}_{{\rm{seg}}},\,{L}_{x})$$

where ${G}_{x}=\{{B}_{x},\,{L}_{x}\}$ are the ground-truth labels for patient detection and skin segmentation labels, respectively, $f(x)=\{{y}_{\det },\,{y}_{{\rm{seg}}}\}$ is the output of the network, ${\alpha }_{\det }$ and ${\alpha }_{{\rm{seg}}}$ are weighting parameters, which are defined based on the relative importance of the patient detection and skin segmentation tasks, respectively, in the unified loss function.

The model was initialised with the original VGG-16’s weights, which hold accumulated knowledge on edges, patterns and shapes learned from the 1.3-million images in the ImageNet dataset.^75^ All the new weight layers, except for the convolutional transpose layers, were initialised using the Xavier algorithm^81^ with zero bias. The Xavier initialisation process created a reasonable range of weight values that were uniformly distributed across the layers. Such an initialisation can lead to faster convergence during training.^81^ The CNN network was implemented within the MatConvNet framework decribed by Vedaldi and Lenc.^82^ The training was performed using standard Stochastic Gradient Descent (SGD) optimisation in two stages. The network was first trained for the skin segmentation task using only the images containing the infant with annotated skin regions. Training was done using the unified loss function (see equation 4) with the parameters ${\alpha }_{\det }=0$ and ${\alpha }_{{\rm{seg}}}=1$. The learning rates were scheduled to start at $1{0}^{-2}$ and reduced by a factor of 10 for every two epochs until convergence, with a momentum of 0.90 and a batch size of 20. These parameters allowed the training and validation losses to reduce gradually and eventually converge to steady values. The network was subsequently trained jointly for the patient detection and skin segmentation tasks using the whole dataset. The individual loss functions for each task were weighted equally: ${\alpha }_{\det }=1$ and ${\alpha }_{{\rm{seg}}}=1$. The learning rate started at $1{0}^{-4}$ and was decreased by a factor of 10 for every two epochs until convergence, with a momentum of 0.90 and a batch size of 20.

### Intervention detection

**Fig. 10:**
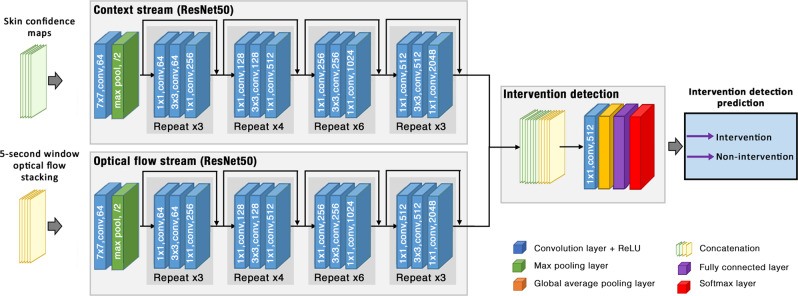
The intervention detection network consists of two input streams operating on 5-second sliding windows. The first input stream (context stream) processed a stack of skin confidence maps, produced by the patient detection and skin segmentation network. The second input stream (optical flow stream) handled a stack of dense optical flow. The outputs from both input streams were then combined to predict the occurrence of a clinical intervention in a given time window.

To detect the occurrence of clinical interventions, the information processed in the patient detection and skin segmentation network was combined with temporal information computed from the optical flow between images in a time window (see Fig. 10). Optical flow comprises a 2-dimensional vector containing the displacements of points between two images in the horizontal and vertical directions.^83^

The original implementation of the two-stream network proposed by^63,84^ computed the optical flow between consecutive frames. Our clinical study consisted of $6\;-\;8$ h per video session and contained over 32.5 million video frames. In order to identify periods of clinical intervention during long video recordings, a sliding-window approach was used to process the video sequence with a fixed window length $T=5$ seconds and a step size $\tau =1$ second, computing the optical flow between images extracted every second. Given a $T$-second sliding window, $L+1$ video frames were extracted, one image every second. Therefore, $L$ optical flows were computed from $L+1$ video frames. The horizontal and vertical components of each optical flow vector were stacked together across input channels, as suggested by Simonyan and Zisserman.^63^

The optical flow network was implemented upon the ResNet50 network with 50 weighted layers, as proposed in ref. ^85^ (see Fig. 10). Even though the number of weighted layers in the ResNet50 network was higher than the VGG16 network used for patient detection and skin segmentation, each layer in the ResNet50 was smaller and had fewer number of parameters. The implementation of the optical flow network using the ResNet50 network allowed the task to be performed with fewer parameters and a lower amount of computational resources than the VGG networks used in refs. ^63,84^ Instead of accepting a single RGB image, the ResNet50 architecture was modified to accept a stack of 2L dense optical flow components. The first convolutional layers were extended to 2L channels by stacking the spatial average of the first convolutional filters across the channels. In order to maintain the aspect ratio of the videos in our clinical study, the network was designed to take a $256\times 192\times 2L$ volume as input. The input size was limited by the computation time and the memory requirements for the workstation used to develop the algorithms. The last fully-connected layer was modified to produce two outputs for the intervention and non-intervention labels, and its filters were re-initialised with the Xavier algorithm with zero bias.^81^ The dropout ratio was changed to 0.85 as suggested in.^84^ The output of this network was the classification score of intervention and non-intervention events.

The training of the intervention detection network required a dataset of annotated intervention periods. To obtain training data, the start and end time points of three mutually exclusive events were annotated in the video dataset: intervention, non-intervention and infant absence. Three human annotators were employed to label the dataset. All video sessions in the 15-infant dataset were annotated. Similar to the training data for the patient detection and skin segmentation network, the periods during which the infant was under phototherapy were excluded from the annotation. The annotators were required to label the periods of intervention, non-intervention and baby absence for a total of 214.0 h of video. A specialised annotation tool was developed. Annotators were asked to watch videos played at 30 times the original speed. This was to ensure that the videos were seen by the annotators in a reasonable amount of time. They could navigate forward and backward in time. Forward navigation was not allowed unless the video section had previously been watched. The annotators were asked to mark the start and end frame numbers in the video for sections during which medical staff or parent(s) were present in the video frame (intervention), the baby was present in the video frame without medical staff or parents (non-intervention) and the baby was not present in the frame (infant absence).

The intervention labels provided by the three annotators were combined based on the consensus among the annotators. Subsequently, the annotations, which were performed at the frame level, were converted into one-second labels using the consensus scores among the labelled video frames for each second.

The Fleiss’ kappa inter-rater reliability of agreement between the three annotators was 96.1%. Of the 214.0 h of annotated videos, 178.9 h were marked as non-intervention, 16.7 h were marked as intervention and 18.4 h were marked as baby absence periods.

We developed three fusion strategies for combining the information from the patient detection and skin segmentation network with temporal information from the optical flow network: (1) spatio-temporal fusion, (2) multi-resolution temporal fusion and (3) temporal context fusion. The spatio-temporal fusion strategy directly combined appearance information extracted from RGB video frames with temporal information extracted from the multiple-frame optical flow network. Several modifications were made to integrate the two networks together. In the patient detection and skin segmentation network, an additional $4\times 4$ max-pooling layer was added after the last convolution layer of the shared core network (see Fig. 9) in order to produce an output with a $8\;\times\;6$ spatial size to match the output of the optical flow network. In the intervention detection branch (see Fig. 10), the outputs from the patient detection and skin segmentation networks and the output from the optical flow network were fused together through a series of concatenation and convolution layers as in ref. ^84^ Concatenation is a process of stacking two or more feature maps with the same spatial size together across channels. Both output feature maps were stacked together and then convolved with a $1\times 1$ convolution layer with 512 feature channels, producing $8\;\times\;6\;\times\;512$ feature maps. The convolution layer performed weighted combinations of spatial and temporal feature maps and reduced the dimension of the combined feature channels. This layer could learn information corresponding to a decision-making process from both networks. The network ended with a global average pooling layer, 2-way fully-connected and softmax layers. The use of global average pooling and fully-connected layers for classification enforces the correspondence between feature maps and categorical outputs.^85^ The last layer provided classification scores to distinguish between non-intervention and intervention events.

Our second fusion strategy, multi-resolution temporal fusion, was based on the network proposed by ref. ^86^ It used two input streams: the main optical flow network which computed optical flows over the entire images (full-frame optical flow); and the local optical flow network that computed optical flows from cropped images containing only the patient area (patient-cropped optical flow). A major advantage was that the multi-resolution fusion could learn temporal information from both global and local contexts. Since the main optical flow network processed a stack of full-frame optical flows and the local optical flow network processed a stack of patient-cropped optical flows, the feature maps from the last convolution layers from both networks were not spatially aligned. Fusion was performed through the concatenation operation, similar to the spatio-temporal fusion, with additional steps to combine two feature maps with different spatial sizes. In the intervention detection branch (see Fig. 9), the output from each network was first passed through a global average pooling layer to reduce its spatial dimension. The output from the average pooling layer was later passed through a fully-connected layer with 512 outputs. Consequently, the outputs from the fully-connected layer of both networks were concatenated across feature channels and then processed through another fully-connected layer with 2 outputs. Finally, the last softmax layer produced classification scores for intervention and non-intervention events.

The third fusion strategy, the temporal context fusion, combined the information from multiple-frame skin confidence maps with multiple-frame dense optical flow. Unlike the two-stream spatio-temporal network, where fusion occurred in the first layers, the temporal context fusion approach used a new context network to process multiple-frame skin confidence maps. These maps were provided by the patient detection and skin segmentation network before being combined with the outputs of the optical flow network. The skin confidence map was defined as the softmax output of the skin segmentation branch of the patient detection and skin segmentation network before applying a threshold to compute the skin and background labels. The skin confidence maps over a video segment contained the information related to the motion of the infant as well as that of the clinical staff, if they were present in the image because an intervention was being performed. The architecture of the context network was similar to that of the optical flow network, but it instead accepted multi-frame skin confidence maps with the same spatial size as the optical flow data ($256\;\times\;192$ pixels). We used the skin confidence maps of the same $L+1$ video frames that were used to compute the $L$ optical flow components. Hence, the context network processed a stack of $L+1$ confidence maps, whereas the optical flow network processed a stack of $2L$ optical flow components. A final decision was made in the intervention detection branch based on the combination of information from both networks. The intervention detection branch was implemented using the convolution fusion approach^84^ similar to that of the two-stream spatio-temporal fusion. The outputs before the global average pooling layer of the context and optical flow networks were fused together through a series of concatenation and convolution layers. The feature maps of both networks were concatenated together along feature channels resulting in $8\times 6\times 4096$ feature maps, followed by a $1\times 1$ convolution layer with 512 outputs. The branch computed a decision on intervention and non-intervention events through a series of global average pooling, 2-way fully-connected and softmax layers.

For each training iteration, video segments were sampled uniformly across intervention and non-intervention classes to create a balanced training set. The training was performed using standard Stochastic Gradient Descent in two stages in order to reduce training time and avoid overfitting. In the first stage, the main optical flow network was trained with a momentum of 0.90 and a batch size of 24 samples. The learning rates were scheduled to start at $1{0}^{-3}$ and reduced by a factor of 10 for every 12,000 iterations until convergence. The same configuration was used for the local optical flow network and the context network. In the second stage, the patient detection and skin segmentation network was integrated with the other network(s), and the intervention detection branch was added to form a fusion network. For each fusion network model, fine-tuning was performed with a momentum of 0.90, a batch size of 12 and a learning rate of $1{0}^{-5}$. The learning rate was decreased by a factor of 10 for every 6000 iterations until convergence. Fine-tuning was performed on only the layers added after fusion, as suggested in refs. ^84,86^

### Evaluation protocol for the CNN models

An approach to obtaining predictive performance is cross-validation using two independent folds. The training set of 15 patients (see Table 2) was firstly divided into two groups, ${D}_{1}$ and ${D}_{2}$, such that one group had eight subjects and the other group had seven subjects. The assignment to different sets was based on a balance of choice between skin phenotype, corrected gestational age and the number of positive images. For each set, positive images were taken directly from the positive pool, and negative images were randomly sampled without replacement from the negative pool so that the number of positive and negative images were equal. A model was first trained on ${D}_{1}$ and validated on ${D}_{2}$. Then, another model was trained on ${D}_{2}$ and validated on ${D}_{1}$. The validation results from both models were combined to produce the overall predictive performance. The main advantage of this approach was that all images were used for both training and validation.

For the patient detection and clinical interventions detection tasks, the classifiers’ performance were described using the Receiver Operating Characteristics (ROC) curve, accuracy, precision, true positive rate (TPR or recall) and true negative rate (TNR or specificity). For the skin segmentation task, a pixel-wise intersection-over union (IOU), which is the standard metric for evaluating a segmentation algorithm, was used to describe the segmentation performance. The IOU metric is defined as:

5 $${\rm{IOU}}=\frac{{y}_{p}\cap {y}_{g}}{{y}_{p}\cup {y}_{g}}$$

where ${y}_{p}$ denotes a predicted segmentation result and ${y}_{g}$ denotes a ground-truth label.

### Reference physiological values

The Philips IntelliVue patient monitoring system used in our study provided two heart rate measurements: one derived from the ECG, recorded by the Philips measurement module; and the other derived from the PPG, recorded by the Masimo pulse oximetry module. Ideally, the monitor would report the same heart rate estimates from the two sources; however, the measurement of a physiological process implies some degree of error. The MAE between both heart rate estimates was 4.1 beats/min with a MAD of 4.0 beats/min. Even though the heart rate estimates from the two devices were highly correlated (correlation coefficient of 0.95), large differences were found across the recording sessions, even when the infants were quiet and had minimal motion.

There are several reasons for the discrepancies between the two sources. Although the Philips IntelliVue monitor is an integrated modular system, each measurement module uses its own internal clock for its acquisition system. Different device manufacturers use their own proprietary algorithms to estimate heart rate, which are usually not disclosed, including different averaging or smoothing techniques. Both manufacturers are compliant with the ANSI/AAMI EC13:2002 “Cardiac monitors, heart rate meters, and alarms standard” standard,^87^ yet the standard only requires the maximum heart rate measurement error to be 1% or 5 beats/min, whichever is greater. There are intrinsic differences between the ECG and PPG signals; the ECG sensor measures the electrical signals generated by the activity of the heart, whereas the PPG measures changes in blood volume underneath the skin. The neonatal population also presents different characteristics in comparison to the adult population that require separate guidelines for the clinical interpretation of the ECG^88^ and PPG.^89^ In our study, the two devices often produced different values during physiological events such as bradycardia or apnoea. Clinical interventions, changing measurement sensors or other motion artefacts occurring when the baby moved a body segment to which a sensor was attached (i.e. legs, arms or upper body), decreased the accuracy of the heart rate estimates.

When two sensing devices are used, neither provides an absolute correct measurement. Since the true value of the heart rate was not known, a direct comparison between the camera-derived heart rate and the heart rate values provided from either of the two reference devices could lead to incorrect performance results. The average of the measurements from two devices or methods is usually taken as the representative values.^90^ Thus, a new robust reference heart rate can be obtained by analysing the agreement between the measurements provided by the two devices. These new gold-standard heart rate values was used to compare the estimates computed from the video camera.

The signal quality of the reference data was not provided by either of the manufacturers. Our proposed process started by identifying periods during which the reference signals were of poor quality by computing Signal Quality Index (SQI) metrics for the ECG and PPG waveforms separately, using established algorithms validated using clinical databases in the public domain. Subsequently, the new reference heart rate estimates were calculated, on a second-by-second basis, as the mean of the two heart rate measurements for which both values did not differ by more than 5 beats/min (as recommended by the ANSI/AAMI EC13:2002 standard^87^), and for which the SQIs of the ECG and PPG were greater than 0.5.

The new reference heart rate was valid for 388.9 h, approximately 91.2% of the total recording time of 426.6 h. The MAE was 0.9 beats/min and MAD was 1.0 beats/min. with a high correlation coefficient of 0.99. Over 200.1 h of 216.6 h (92.5%) were found to be valid in the training set. Similarly, over 188.6 h of 210.0 h (89.8%) were valid in the test set. These results imply a good agreement between the ECG heart rate and PPG heart rate estimates under the conditions described above. A detailed discussion on the analysis of the estimates from the reference devices can be found at refs. ^65,66^

The Philips IntelliVue patient monitor used in our study provided respiratory rate estimates derived from the IP signal using proprietary algorithms, without a corresponding quality measure. The IP signal is known to be affected by noise and artefacts, which could lead to errors in the estimation of respiratory rate.^91,92^ In sick newborn infants, the movement of the upper body during active awake periods often causes large motion artefacts in the IP signal, which prevents a reliable estimation of respiratory rate.^92,93^ The shallow and irregular breathing patterns of preterm infants make it difficult to measure respiratory rate using patient monitoring equipment. In our dataset, large discrepancies were found when comparing the respiratory rate reported by the patient monitor with that computed by manual breath counting by the trained clinical staff.^94,95^ The respiratory rates provided by the patient monitor were not suitable to be used as reference values for comparing with camera-derived estimates. Therefore, a new gold-standard reference respiratory rate was needed.

Using the algorithms to assess the quality of the ECG waveform, performed as part of the estimation of the reference heart rate, our proposed system started by extracting three respiratory signals from the ECG: ECG-derived respiration (EDR), respiratory sinus arrhythmia (RSA) and R-peak amplitude (RPA). With the addition of the IP waveform, SQI metrics were computed for the four respiratory signals using well-known algorithms that have been extensively validated by the research community on publicly available physiological databases. Subsequently, respiratory rate was estimated from each signal using two methods: a time-domain technique, by counting the number of breaths within a window; and a frequency-domain method, by finding the frequency of the dominant pole of an AR model. Two new respiratory rate estimates were computed, one for each method, by combining the individual respiratory rates for each individual signal with a data fusion algorithm. Finally, the new reference respiratory rate was computed as the mean of the combined respiratory rate estimates from both methods during which the data were of good quality and their difference was less than 5 breaths/min. The maximum error of 5 breaths/min has been used as a primary outcome measure in many clinical trials involving the measurement of respiratory rate that have been approved by the U.S. National Institutes of Health (NIH).

The new reference respiratory rate was valid for over 189.0 h, approximately 44.3% of the total recording time of 426.6 h. The MAE was 2.2 breaths/min and MAD as 1.4 breaths/min, with a high correlation coefficient of 0.98. Over 95.3 h of 216.6 h (44.0%) were found to be valid in the training set. Similarly, over 93.7 h of 210.0 h (44.7%) were valid in the test set. The results are consistent with the values published in the literature that vary between 29%^91,96^ and 32%.^66^ A detailed discussion on the analysis of the estimates from the reference devices can be found at refs. ^65,66^

### Heart rate estimation

The framework presented in the previous sections provided accurate per-frame skin segmentation from which a region of interest could be selected to extract a PPGi signal and to compute heart rate estimates. The PPGi signal contains cardiac pulsatile AC variations superimposed on a non-pulsatile DC component. Tarassenko et al.^43^ showed that a skin ROI needs to be large enough such that a PPGi signal with a strong cardiac component can be obtained. The raw PPGi signal was derived by spatially averaging all pixels in the whole skin area for the green colour channel for each frame in the video. The raw PPGi signal was extracted from the raw uncompressed video data stored at a resolution of $1620\times 1236$ pixels and at a rate of 20 frames per second. Let ${G}_{t}$ be the green channel of a video frame ${I}_{t}$ at time $t$ and ${S}_{t}\in \{0,1\}$ be a skin label of ${I}_{t}$, where the subscripts 0 and 1 denote non - skin and skin classes, respectively. The raw PPGi signal was defined as:

6 $${{\rm{PPGi}}}_{{\rm{raw}}}=\frac{1}{{N}_{{\rm{skin}}}}\sum _{i=1}^{{N}_{x}}\sum _{j=1}^{{N}_{y}}{G}_{t}(i,j){S}_{t}(i,j)$$

where $i$ and $j$ are spatial coordinates, ${N}_{x}$ and ${N}_{y}$ are the number of rows and columns in the image, respectively, and ${N}_{{\rm{skin}}}$ is the number of skin pixels in the image. The raw PPGi signal was computed on a per-frame basis such that the signal’s sampling rate was equal to the video frame rate of $20$ Hz.

The PPGi signal contained pulsatile components correlated with the cardiac frequency as well as motion artefacts and often other sources of noise. Since the raw PPGi signal extracted from the skin was sensitive to disturbances such as motion artefacts and lighting changes, the signal was first detrended in order to remove any non-pulsatile DC offset, and then filtered to reduce frequency components outside the physiological range of interest. The normal heart rate of preterm infants ranges from $90$ to $180$ beats/min,^67^ which is higher than that of healthy adults and full-term infants. Analysis of the reference heart rates in the training set showed that more than 99% of the values were concentrated in the range of 90–270 beats/min (1.5–4.5 Hz). Therefore, the raw PPGi signal was processed with a cascade of a 40${}^{th}$-order low-pass Finite Impulse Response (FIR) filter with a cut-off frequency at 4.5 Hz and a 60${}^{th}$-order high-pass FIR filter with a cut-off frequency at 1.5 Hz.

A peak and onset detection algorithm based on Zong et al.^97^ and later extended by Villarroel et al.^66^ was then applied to the PPGi signal to identify salient points for each heart beat. The algorithm was modified for the neonatal population by defining the duration of the upslope of the pulse as a window of 150 ms, corresponding to three samples for a PPGi signal with a sampling frequency of 20 Hz. The algorithm was shown to be effective in detecting the peaks and onsets o pulsatile signals.^66,97,98^

Accurate peak and onset detection allowed the beat-by-beat assessment of the quality of the PPGi signal. As an initial step, an activity index was computed based on changes in the segmented skin area over consecutive frames, corresponding to the movement of the subject. A Bayesian change point detection algorithm was then applied to identify step changes in the PPGi signal, often caused by sudden lighting condition changes. The pulses occurring during the periods of high subject motion and step changes were flagged as invalid. In order to further identify whether each detected beat was of good quality, the algorithm performed a beat-by-beat quality assessment by combining multiple analysis methods: frequency bounding, clipping detection, amplitude thresholding and multi-scale dynamic time warping. Finally, the SQI of each detected beat was obtained as a combination of all these individual metrics. The derivation of the SQI values for heart rate estimation can be found in the supplementary information 4 provided for this paper.

Heart rate was computed using a running window of 8 seconds with a step size of 1 second. The SQI value for each heart rate estimate was calculated as the mean of the beat-by-beat SQI values for the respective 8-second window. Heart rate estimation was performed using four algorithms: beat counting, Fast Fourier transform (FFT), dominant pole of an autoregressive model (AR dominant pole), and choosing the best model order of multiple autoregressive models (AR best model).

To count the number of beats, the time window $w$ of 8 seconds was first expanded to include the peaks of the first and last beats. Heart rate was then computed as:

7 $$HR(w)={N}_{{\rm{beats}}}\cdot \frac{60}{{L}_{\exp }}$$

where ${N}_{{\rm{beats}}}$ is the number of beats in the expanded window and ${L}_{\exp }$ is the length of the expanded window.

The second method computed heart rate by identifying the dominant frequency with the highest power in the Fast Fourier Transform (FFT) of the PPGi signal. The heart rate estimates obtained from the FFT method were affected by quantisation errors since the frequency resolution of the FFT depends on the sampling rate (${f}_{s}$) and the number of samples used to compute the FFT.

Autoregressive (AR) modelling was also used to identify frequency content in the PPGi signal. Unlike the FFT technique, the AR model has no frequency resolution limitations when applied to short-time series segments. Heart rate was calculated by finding the dominant pole that was located inside the angle (in radians) of interest, corresponding to a heart rate range between 90 and 270 beats/min, in the $z$-transform of the AR model. The choice of model order was a compromise between a higher model order which can provide a better approximation but can also fit the noise in the signal, and a lower model order, which may not be sufficient to represent the signal.^99^ The algorithm used a fixed model order of 8, which was found to achieve the lowest mean absolute error in the training set.

A further algorithm to estimate heart rate from the PPGi signal was implemented to choose the best model order in a range between 6 and 12. The choice of the best model order was made by comparing the frequency of the dominant pole and the frequency of the highest peak of the frequency response of the model. The frequency response of an AR model of order $p$ and noise variance ${\sigma }_{e}^{2}$ is given by:^99^

8 $$S(f)=\frac{{\sigma }_{e}^{2}}{| \sum _{k=0}^{p}{a}_{k}{e}^{-i2\pi fk}{| }^{2}}$$

where ${a}_{k}$ are the coefficients of the AR model. The best model was computed as the model for which the difference between the frequency of the dominant pole and the frequency corresponding to the highest peak of the frequency response (calculated using equation 8) in the frequency band between 1.5 and 4.5 Hz was less than 1 beat/min. If more than one model order met this criterion, the model with the highest amplitude of the dominant pole was chosen. If no model met this criterion, the heart rate for that time window was estimated by finding the highest peak of the frequency response, as described by equation (8).

Once heart rate had been computed for every 8-second window, a Kalman filter was applied similarly to refs. ^100,101^ The heart rate estimates were adjusted based on their signal quality, reducing the effects of transient changes of noise and motion artefacts.

### Respiratory rate estimation

During each respiratory cycle, the infant’s chest and abdomen expand and contract with breathing. This phenomenon causes movement of the body that can be recorded by a video camera from areas containing exposed skin or covered by tight-fitting clothing such as a nappy. The CNN for skin segmentation was used as the first step in the extraction of twelve respiratory signals divided in three groups. The first group consisted of three respiratory signals derived from the PPGi signals extracted from each of the video camera’s three colour channels. The second group comprised four respiratory signals extracted from four properties of the patient’s segmented skin area. The last group consisted of five respiratory signals extracted from geometrical properties of an ellipse fitted to the skin area.

The PPGi signal, computed by averaging the colour over the skin regions, contained both cardiac and respiratory information. The relative size of the respiratory-correlated pulsatile component in the PPGi signal depended on the gestational age (reflecting the size and developmental stage of the infant) and the breathing pattern (shallow or deep breathing). Three PPGi signals were extracted from the skin regions: $PPG{i}_{{\rm{red}}}$, $PPG{i}_{{\rm{green}}}$ and $PPG{i}_{{\rm{blue}}}$ derived from the red, green and blue colour channels respectively.

The contraction and relaxation of the muscles during respiration causes the volume of the chest cavity to increase or decrease, resulting in motion of the chest and abdomen.^102^ Respiratory signals can be acquired by tracking these motion changes across the subject’s skin areas.^103^ Four respiratory signals were extracted by computing the following shape properties from the entire skin label for each video frame: area, perimeter and the $x$ and $y$ coordinates of the centroid.^104^

Although changes in the skin region properties over time could reflect the motion of the subject, which in turn could be used to estimate respiratory rate, small degrees of non-respiratory motion easily introduced motion artefacts and corrupted the respiratory signals. Similarly, the subject’s posture and clothing sometimes split the skin into smaller regions, so the respiratory signal extracted from the properties of multiple skin regions could contain abrupt changes from motion artefacts as well. The result of skin segmentation often has an elliptical shape (see Fig. 3). Changes in the shape of the ellipse over time as a result of breathing could be used to extract a respiratory signal. If there were more than one separate non-contiguous skin region, the upper body area, which had a prominent respiratory motion usually corresponded to the largest continuous region.^103^ Therefore, for each video frame, an ellipse was fitted to the largest continuous skin region by matching the second central moments of the skin region to the ellipse. Five respiratory signals were then extracted from changes in: major axis length, minor axis length, orientation, eccentricity and elliptical area.^104^

The respiratory signals extracted from the video data were inherently noisy and were often contaminated by baseline drifts and high-frequency noise. To remove these artefacts, each of the twelve respiratory signals was detrended and filtered using a cascade of a $10{0}^{th}$-order high-pass FIR filter with a cut-off frequency at 0.3 Hz and an $8{0}^{th}$-order low-pass FIR filter with a cut-off frequency at 2.0 Hz. These filters encompassed respiratory rates in the range of 18–120 breaths/min.

Once the respiratory signals had been extracted, two algorithms for peak and onset detection were applied: the first one was based on the mean average curve (MAC)^105,106^ and the second was based on the boxed slope sum function (BSSF).^97^ Originally, the BSSF algorithm was designed for detecting peaks and troughs in a cardiac signal. Several parameters were changed to make the algorithm work with a respiratory signal. Instead of using the typical duration of the upslope of the cardiac pulse for computing the BSSF signal, the upslope duration of the respiratory pulse was modified to 300 ms. To prevent multiple detections, a 500 ms refractory period was applied. Both detection algorithms have high accuracy but different sensitivities to different types of noise.^101^

The amplitude of each breath pulse generally depended on how much the skin colour changed or how much the subject moved during breathing. During a quiet and stable period, the amplitude of each peak generally reflected the depth of each breath: shallow breathing resulted in a low-amplitude waveform, while deep breathing resulted in a high-amplitude waveform. Preterm infants generally have different breathing patterns than those from term infants and adults as a result of weak ribs, weak muscles, lack of surfactant (substance in the lungs that facilitates gas exchange) and low respiratory effort.^107^

Respiration was more prominent when the subject was quiet with minimal body motion. The primary aim of the signal quality assessment was to provide a quality measure from 0 (poor quality) to 1 (good quality) for each breath. For each of the twelve respiratory signal extracted, four signal quality indices were computed based on the analysis of the patient activity, a valid physiological breathing range, the agreement between peak detectors and multi-scale dynamic time warping. The calculation of the first two SQIs followed what was described in the previous section for heart rate estimation. The derivation of the SQI for respiratory rate estimation can be found in the supplementary information 5 provided for this paper.

Following the signal quality assessment, the respiratory rate for each of the twelve respiratory signals was estimated using a 10-second sliding window with a step size of 1 second. Respiratory rate was therefore reported every second. Prior to the estimation, the time window was expanded to include the complete first and last breath pulses. Estimation was performed by counting the number of breaths over the time window. The final signal quality index of the respiratory rate was calculated as the mean of the SQI values over the given window when the numbers of breaths detected by both detectors were equal. When the numbers of breaths detected by both detectors were not equal, the SQI was taken to be 0, corresponding to a poor-quality window.

Once respiratory rate had been estimated for all respiratory signals, a data fusion technique based on multiple Kalman filters (as for heart rate estimation) was applied to combine the multiple respiratory estimates from the same time window and produce a final respiratory rate and a final signal quality index.

### Reporting summary

Further information on research design is available in the Nature Research Reporting Summary linked to this article.

## Supplementary information

Reporting Summary

Supplementary Material

## Data Availability

The datasets generated during and/or analysed during the current study are not publicly available due to the sensitive and identifiable nature of our data, parental consent and restrictions of the ethics protocol to protect the privacy of preterm infants involved in the study.

## Code availability

Two open-source custom software packages are available at:^69^ code for the semi-automatic labelling of skin regions of people in images, and software for annotating the time periods during which clinical interventions occur in videos.
