# Supplementary material for: Non-contact physiological monitoring of preterm infants in the Neonatal Intensive Care Unit
Source: NPJ Digit Med. 2019 Dec 12;2:128. doi: 10.1038/s41746-019-0199-5 (PMC6908711; doi:10.1038/s41746-019-0199-5)
Supplement: Supplementary file 2 — Supplementary Material [file 41746_2019_199_MOESM2_ESM.pdf]

Non-contact physiological monitoring of preterm infants in the Neonatal Intensive Care Unit

Mauricio Villarroel[1,*], Sitthichok Chaichulee[1], João Jorge[1], Sara Davis[2], Gabrielle Green[2], Carlos Arteta[3], Andrew Zisserman[3], Kenny McCormick[2], Peter Watkinson[4], Lionel Tarassenko[1]

1 Institute of Biomedical Engineering, Department of Engineering Science, University of Oxford, UK
2 Neonatal Unit, John Radcliffe Hospital, Oxford University Hospitals Trust, UK
3 Visual Geometry Group, Department of Engineering Science, University of Oxford, UK
4 Nuffield Department of Clinical Neurosciences, University of Oxford, UK
* mauricio.villarroel@eng.ox.ac.uk

Supplementary material

Supplementary method 1: Patient detection and skin segmentation performance

The proposed CNN networks for patient detection and skin segmentation were developed and evaluated with a two-fold cross validation procedure using images extracted only from the 15 preterm infants dataset labelled as "training" in table 2 of the main paper. The proposed CNN models were compared with the performance of three colour-based skin classifiers[1,2] based on Naive Bayes[3], Random Forests[4] and Gaussian Mixture Models (GMMs)[5]. The skin filters classify each pixel as a skin pixel based solely on skin colours and provide a skin probability map, which can be thresholded to a binary label. The skin models were trained on images that were converted to the Hue-Saturation-Lighting (HSL) colour space[6] with white balance correction applied[7,8]. Patient detection was performed using the ratio of skin to non-skin pixels and the average probability of predicted skin pixels to make a decision, as in the method described in[3].

Supplementary table 1. Patient detection performance of the baseline skin filters and the proposed CNN models.

Model	AUC	Accuracy	Precision	Recall	Specificity
Baseline skin filters					
Naive Bayes	98.1	98.6	**97.8**	99.4	**97.8**
Random Forests	97.2	97.7	97.5	97.9	97.4
GMMs	98.8	97.1	97.8	96.4	**97.8**
CNN without data augmentation					
CNN patient detection only	97.7	98.0	96.1	**100.0**	96.0
Multi-task CNN	**99.7**	98.2	96.6	**100.0**	96.5
CNN with data augmentation					
CNN patient detection only	97.9	97.1	96.0	98.3	95.4
Multi-task CNN	98.2	**98.8**	97.6	**100.0**	96.8

All values are expressed as a percentage.

Supplementary table 1 shows the results for the patient detection network compared with the baseline skin filters. The dataset without data augmentation consisted of a total of 3,436 images divided in 1,718 positive images (with an infant in the video frame) and 1,718 negative images (without an infant in the video frame). As explained in the "Methods" in the main paper, multiple variations of each image were generated using three data augmentation techniques: rotational, mirroring and lighting augmentation. The total number of the dataset with data augmentation was 44,668 divided in 22,334 positive and 22,334 negative images. The datasets were split equally between the training and test sets. The Naive Bayes classifier achieved the highest accuracy in the patient detection task among the baseline skin filters. It achieved 1.0% and 1.6% higher accuracy than Random Forests and GMMs respectively. In term of the area under the receiving operating curve (AUC), GMMs obtained the highest score (98.8%) followed by Naive Bayes (98.1%) and Random Forests (97.2%) respectively.

Supplementary table 2. Skin segmentation performance of the baseline skin filters and the proposed CNN models.

Model	Pixel Accuracy			Intersection over Union		
	Mean (SD)	Min	Max	Mean (SD)	Min	Max
Baseline skin filters						
Naive Bayes	89.5 (8.3)	32.7	98.9	61.3 (17.4)	4.3	92.9
Random Forests	95.0 (4.6)	57.7	99.3	75.9 (16.1)	6.8	95.4
GMMs	93.4 (5.2)	47.5	99.1	71.2 (14.2)	16.8	94.7
CNN without data augmentation						
CNN skin segmentation only	92.2 (3.4)	71.1	74.4	57.4 (15.2)	0.00	84.5
Multi-task CNN	96.2 (2.0)	**75.9**	98.9	77.2 (9.9)	4.8	92.9
CNN with data augmentation						
CNN skin segmentation only	97.9 (1.2)	88.7	99.5	87.8 (6.0)	**49.4**	96.5
Multi-task CNN	**98.1 (1.9)**	75.6	**99.6**	**88.6 (7.5)**	39.0	**97.0**

All values are expressed as a percentage.
Performance evaluated only on positive images with the presence of a subject.

Skin segmentation was performed considering only the positive images with an infant present in the video frame. Supplementary table 2 shows the results for the proposed skin segmentation networks. The dataset without data augmentation consisted of 1,718 images, the dataset with data augmentation consisted of 22,334 images. Random Forests achieved the best performance for skin segmentation, with 4.7% and 14.5% improvements in intersection-over-union (IOU) with respect to GMMs and Naive Bayes respectively.

The multi-task CNN model trained with data augmentation outperformed the other models for the majority of the metrics in both patient detection and segmentation tasks. For patient detection, the model achieved an accuracy of 98.8% and an AUC score of 98.2%. For skin segmentation, the network yielded an IOU score of 88.6% and a pixel accuracy of 98.1%.

Supplementary method 2: Intervention detection performance

Time periods of clinical intervention were detected by combining information processed in the patient detection and skin segmentation network with temporal information computed from the optical flow between images over a sliding time window of length T and step size τ. For each T-second sliding window, L optical flows were computed from $L+1$ video frames, each one extracted every second. The horizontal and vertical components of each optical flow vector were stacked together across input channels, as suggested by Simonyan and Zisserman[9]. The proposed clinical intervention network was developed and evaluated with a two-fold cross validation procedure using only the 15 preterm infants dataset labelled as "training" in table 2 of the main paper, as stated by the clinical study protocol.

Supplementary table 3. Baseline performance of the two-stream architecture proposed by[9] evaluated using a window length $T = 10$ seconds and a step size $\tau = 1$ second.

Model	AUC	Accuracy	Precision	Recall	Specificity
Spatial	84.1	76.4	77.0	75.2	77.5
Temporal, $L = 10$	95.3	87.8	84.1	93.3	82.4
Fusion network (Average)	95.4	89.3	86.3	93.4	85.2
Fusion network (SVM)	**98.1**	**92.4**	**90.8**	**94.4**	**90.5**

All values expressed as a percentage.

Supplementary table 3 shows the performance of the baseline implementation compared with reference methods developed based on the two-stream convolutional architecture for action recognition proposed by Simonyan and Zisserman[9] and implemented using the VGG-M-2048 model[10]. As suggested in[9], the number of optical flow stacks L was set to 10. To be comparable with the original implementation, we used a window length $T = 10$ seconds and a step size $\tau = 1$ second. There were a total of 129,608 windows, split equally on windows during which a clinical intervention occurred and windows during which there were no clinical interventions. The results were consistent with those reported in[9]. The temporal network yielded higher accuracy (87.8%) than the spatial network (76.4%). The fusion of both networks, using either averaging or a linear Support Vector Machine (SVM), led to further improvement in the accuracy since they provided complementary information to support the classification task. The SVM fusion network resulted in the highest accuracy of 92.4%, a 4.7% improvement over the temporal network.

Supplementary table 4. Performance of the optical flow network with different sliding window configurations.

Window configuration	AUC	Accuracy	Precision	Recall	Specificity	Total number of windows
$T = 1$ sec. , $\tau = 1$ sec.	95.2	88.5	89.3	87.5	89.5	113,610
$T = 5$ sec. , $\tau = 1$ sec.	**97.4**	**92.2**	**91.7**	**92.8**	**91.6**	121,426
$T = 5$ sec. , $\tau = 5$ sec.	95.9	89.7	90.6	88.5	90.8	24,302
$T = 10$ sec., $\tau = 1$ sec.	96.2	88.2	88.2	91.5	87.8	129,608
$T = 10$ sec., $\tau = 10$ sec.	95.5	88.5	87.2	90.4	86.7	13,006

All values expressed as percentage.

Supplementary table 4 summarises the performance effects of using different sliding window configurations on the optical flow network. The number of windows were different according to each configuration. If more than half a time window was labelled as intervention by the annotators, the whole time window was marked as intervention. Therefore, there was more training data available for longer windows even though the step size is the same (for example $T = 1$ sec, $\tau = 1$ sec compared with $T = 10$ sec, $\tau = 1$ sec). The dataset was split equally on windows during which a clinical intervention occurred and windows during which there were no clinical interventions. The configuration of a 5-second sliding window with 1-second step size led to 92.2% accuracy and outperformed the other configurations. Increasing the size of the window length from 5 to 10 decreased performance.

Supplementary table 5 reports the performance of the local temporal network and the context network that were trained individually and used for constructing the multi-resolution and temporal context fusion networks. These networks were evaluated using a dataset containing a total of 121,426 time windows of length 5 seconds and a step size of 1 second, which was the best performing configuration for the optical flow network, as reported in supplementary table 4.

Supplementary table 5. Performance of the local optical flow network and context network evaluated using a window length $T = 5$ seconds and a step size $\tau = 1$ second.

Model	AUC	Accuracy	Precision	Recall	Specificity
Local optical flow network					
Fit cropping	96.6	90.5	89.8	91.4	89.6
Centre cropping	96.8	91.1	92.9	89.0	93.2
Context network					
Skin heatmap stacking	95.9	89.7	89.6	89.9	89.5

All values expressed as a percentage.

Supplementary table 6 reports the classification performance for different fusion strategies evaluated using a dataset containing a total of 121,426 time windows of length 5 seconds and a step size of 1 second. The temporal context fusion method yielded the highest performance with an accuracy of 94.5%, a 2.3% improvement with respect to the optical flow network alone. Multi-resolution temporal fusion, with either fit or centre cropping, gave marginal performance improvements. In contrast, the spatio-temporal fusion method was unable to make effective use of spatial information extracted through the patient detection and skin segmentation network.

Supplementary table 6. Performance of the different fusion approaches evaluated using a window length $T = 5$ seconds and a step size $\tau = 1$ second.

Model	AUC	Accuracy	Precision	Recall	Specificity
Spatio-temporal fusion					
Single frame	97.0	91.7	89.8	94.1	89.3
Multiple frames	96.0	90.5	90.4	90.8	90.3
Multi-resolution temporal fusion					
Fit cropping	97.8	93.7	92.8	93.7	92.7
Centre cropping	97.7	92.9	94.5	91.1	94.7
Temporal context fusion					
Skin heatmap stacking	98.2	**94.5**	**94.4**	94.7	94.4

All values expressed as a percentage.

Supplementary method 3: Typical clinical interventions and nursing activities in the NICU

Preterm infants experience routine clinical interventions several times a day in the Neonatal Intensive Care Unit (NICU). For example: checking the normal functionality of medical equipment, changing a nappy, taking temperature readings, administering medications or withdrawing blood from the heel for a blood gas test. During these events, clinical staff or parents actively interact with the infant, causing motion artefacts that pose challenges to the estimation of vital signs from video camera data. Supplementary table 7 summarises the activities carried out by the nurses in the care of the pre-term infants in the NICU. Parents also visit their newborn baby regularly and often take the infant from the incubator for kangaroo care (skin-to-skin contact with the parent).

Supplementary table 7. Typical daily nursing activities for pre-term infants in the NICU (Data provided by research nurses at the John Radcliffe Hospital).

Frequency	Event
At nurse shift handover (every 8−12 hours)	– Lift incubator cover to examine the infant.<br>– Examine nasogastric tube (NGT) placement.<br>– Examine central venous line (CVL).
As required	– Check emergency equipment.<br>– Check ventilation equipment.<br>– Check fluid infusion pumps.<br>– Replace electrocardiogram (ECG) leads.<br>– Replace nasal probes.
After bradycardia, O_2 desaturation or apnoea	– Provide tactile stimulation.<br>– Change infant position.
Every hour	– Remove fluid from the airways.<br>– Give nasogastric tube (NGT) feed.<br>– Record vital sign parameters.
Every 6 hours	– Take temperature and blood pressure.<br>– Change skin probe sites.<br>– Change infant position.
Every 12 hours	– Take infant out of incubator for cuddles.
Every 6−8 hours, if under phototherapy<br>Every 2−6 hours, if hypoglycemic	– Heel prick for a blood test.
Every 6−12 hours	– Give oral medication.
Every 4−12 hours	– Give intravenous (IV) line medication.

Supplementary method 4: Signal Quality Index for heart rate estimation

The assessment of the quality of the PPGi signal is of high importance as data corruption by subject movements and changes in the lighting conditions presented considerable challenges for video analysis. The assessment of the quality of the PPGi signal was extended from that described in[11,12] and further described in[13]. As an initial step, an activity index was computed based on changes in the segmented skin area over consecutive frames, corresponding to the movement of the subject. A Bayesian change point detection algorithm was then applied to identify step changes, often caused by sudden lighting condition changes, in the PPGi signal. The pulses occurring during the periods of high subject motion and step changes were flagged as invalid. In order to further identify whether each detected beat was of good quality, the algorithm performed a beat-by-beat quality assessment by combining multiple analysis methods: frequency bounding, clipping detection, amplitude thresholding, and multi-scale dynamic time warping. Finally, the signal quality index (SQI) of each detected beat was obtained as a combination of all these individual metrics.

Suitable time periods for estimating heart rate from the video camera were defined when the movement of the infant was minimal. Changes in the segmented skin area over time can be used as an indicator of the degree of subject motion. The centroid (C_x, C_y) of skin regions was defined as the average location of the predicted skin pixels in the horizontal and vertical directions. Motion $M(i)$ at frame i was defined as the Euclidean distance between centroids for two successive frames:

$$\mathrm{M}(i) = \sqrt{\left(C_x(i) - C_x(i-1)\right)^2 + \left(C_y(i) - C_y(i-1)\right)^2}. \tag{1}$$

The SQI_{act} of the kth beat was taken to be 0 if the Euclidean distance between the kth beat and two beats both before and after (5 beats in total) was higher than a threshold of 20 pixels, defined as:

$$SQI_{\text{act}}(k) = \begin{cases} 0 & \text{if } \exists\, i \in \{b_{k-2}, ..., b_{k+2}\} \ \mathrm{M}(i) > 20 \\ 1 & \text{otherwise} \end{cases} \tag{2}$$

where b_{k-2} and b_{k+2} denote the location of the entire $(k-2)$th and $(k+2)$th beat respectively. The distance threshold was set to 20 pixels, corresponding to a distance of approximately 1 cm measured by the ruler in the colour chart placed near the subject (see figure 1c in the main paper). The camera was positioned approximately 30 cm away from the subject for all recording sessions.

Abrupt changes in the PPGi signal occurred due to changes in subject posture or sudden changes in the lighting conditions, for example: when the overhead light over the incubator was turned on or off; window blinds were opened or closed; or clinical staff walked pass by the incubator. In order to detect the location of these step changes, a Bayesian change point detection algorithms was applied to the PPGi signal[14]. Change point detection was performed on a window-by-window basis for different window sizes of 5, 10 and 15 seconds and a step size of 5 seconds. All the change points detected were then merged together. Given $P_{\text{all}}(m)$ is the probability of a change point at m merged from the detections at multiple window sizes, the SQI_{cp} of the kth beat was defined as:

$$SQI_{\text{cp}}(k) = \begin{cases} 0 & \text{if } \exists\, i \in \{b_{k-2}, ..., b_{k+2}\} \ P_{\text{all}}(i) > 0.50 \\ 1 & \text{otherwise} \end{cases} \tag{3}$$

where b_{k-2} and b_{k+2} denote the location of the $(k-2)$th and $(k+2)$th beats respectively. If a change point was detected at the location of the kth beat, the SQI_{cp} values of this beat and the two beats before and after were set to zero.

Frequency bounding determined whether the instantaneous HR fell within the physiological range of typical preterm infants, taken to be within a range of 90 and 270 beats/min. Given that HR_{inst} is the instantaneous heart rate of the kth beat, the SQI_{freq} was taken to be 0 if the HR_{inst} fell outside the valid physiological range:

$$SQI_{\text{freq}}(k) = \begin{cases} 0 & \text{if } HR_{\text{inst}}(k) < 90 \text{ and } HR_{\text{inst}}(k) > 270 \\ 1 & \text{otherwise} \end{cases}. \tag{4}$$

Clipping generally occurred as a result of motion artefacts. Signal clipping can be detected when the derivative of the signal crosses a given threshold[15]. Given that $N_{\text{length}}(k)$ is the length of the kth beat and $N_{\text{clipped}}(k)$ is the proportion of the derivative

of the kth beat that crosses a clipping threshold of 0.1, the SQI_{clip} of the kth beat was set to 0 when more than one-third of the derivative was clipped:

$$SQI_{\text{clip}}(k) = \begin{cases} 0 & \text{if } N_{\text{clipped}}(k)/N_{\text{length}}(k) > 1/3 \\ 1 & \text{otherwise} \end{cases}. \tag{5}$$

Amplitude thresholding was performed to determine whether the amplitude of each beat remained within three standard deviations σ_w from the mean μ_w of the window w. The statistics were calculated locally for each 15-second moving window w. The SQI_{amp} of the kth beat at location b_k was set to 0 if part of the beat was outside the valid range:

$$SQI_{\text{amp}}(k) = \begin{cases} 0 & \text{if } \exists i \in \{b_k\} \quad PPG_{\text{filt}}(i) > \mu_w + 3 \cdot \sigma_w \text{ or} \\ & \qquad\qquad\quad PPG_{\text{filt}}(i) < \mu_w - 3 \cdot \sigma_w \\ 1 & \text{otherwise} \end{cases} \tag{6}$$

Another quality metric was defined by measuring the similarity of the cardiac beats in the PPGi signal. Dynamic time warping (DTW) is a time series technique used to determine a distance (or a degree of similarity) between two given time series based on the best possible alignment between the two[16]. The DTW technique is suitable for the time series whose characteristics may vary in time. For example, similarities in each cardiac cycle could be measured using the DTW technique, even if heart rate was increasing or decreasing during the course of an observation. Each cardiac beat pulse can be warped in the time domain to determine a degree of similarity, independent of temporal variations. The classical DTW algorithm is computationally intensive as it needs to evaluate every possible warping path in order to obtain an optimal alignment. Fitriani[17] and Salvador[18] extended the algorithm to perform multi-scale warping by refining the search space for the optimal alignment between the two time series from a coarse to a finer resolution. The multi-scale DTW technique was extended for assessing the quality of pulsatile signals by determining the distance of the optimal alignment between each beat and a running beat template computed over a time window.

To compute the signal quality based on the DTW method (SQI_{dtw}), the PPGi signal was first divided into 15-second moving windows with a step size of 5 seconds. Each window was assessed independently of each other. Multi-scale DTW is described in more detail in[11]. The DTW distance was computed between each individual beat (X_k) and the average beat within the window Y_k. The SQI_{dtw} was defined as:

$$SQI_{\text{dtw}}(k) = 1 - DTW(X_k, Y_k)/100. \tag{7}$$

SQI_{dtw} ranges from 0 to 1, where a high value relates to a good-quality beat. On the training set, DTW values for good-quality beats ranged between 5 and 20 with SQI_{dtw} values greater than 0.80. On the contrary, poor-quality beats had much higher $DTYW$ distances and corresponding lower SQI_{dtw} values.

Once all the individual SQI metrics for each beat had been calculated, the combined beat SQI (SQI_{beat}) for the kth beat was derived by simply multiplying all the SQI metrics together:

$$SQI_{\text{beat}}(k) = SQI_{\text{act}}(k) \cdot SQI_{\text{cp}}(k) \cdot SQI_{\text{freq}}(k) \cdot SQI_{\text{clip}}(k) \cdot SQI_{\text{amp}}(k) \cdot SQI_{\text{dtw}}(k). \tag{8}$$

Supplementary method 5: Signal Quality Index for respiratory rate estimation

The signal quality assessment employed a series of different measures to assign a signal quality index to each breath in the respiratory signal. The algorithms presented in this appendix were extended from that described in[11,19] and further described in[13]. Four signal quality indices were computed based on the analysis of the patient activity (SQI_{act}), a valid physiological breathing range (SQI_{freq}), the agreement between peak detectors (SQI_{peak}) and multi-scale dynamic time warping (SQI_{dtw}). The calculation of the first SQI followed what was described in the previous section for heart rate estimation.

Frequency bounding determined whether the instantaneous RR fell within the physiological range of typical preterm infants, taken to be within a range of 18 and 120 breaths/min. Given that RR_{inst} is the instantaneous respiratory rate of the kth breath, the SQI_{freq} was taken to be 0 if the RR_{inst} fell outside the valid physiological range:

$$SQI_{\mathrm{freq}}(k) = \begin{cases} 0 & \text{if} \quad RR_{\mathrm{inst}}(k) < 18 \ \text{ and } \ RR_{\mathrm{inst}}(k) > 120 \\ 1 & \text{otherwise} \end{cases}. \tag{9}$$

Unlike the estimation pipeline used for heart rate estimation, the assessment of quality of the respiratory signals was based on the agreement between two peak and onset detection algorithms[20]. Peak agreement is a measure of how much the peaks identified by the two peak-and-onset detectors agreed with each other over a given time window. Both detectors usually agreed with each other when the respiratory signal was clean and disagreed in the presence of noise and artefacts. Agreement was considered valid when the peaks identified by the two detectors were not located away from each other by more than 5 samples (or 0.25 seconds – half of the duration of the highest frequency rate that could be estimated). The measure of peak agreement SQI_{peak} for the kth breath was calculated as the ratio of the number of peaks in agreement over the total number of peaks detected in a 10-second window, centred around the kth breath:

$$SQI_{\mathrm{peak}}(k) = \frac{N_{\text{Agreed peaks}}}{N_{\text{All peaks}}} \tag{10}$$

The multi-scale dynamic time warping technique, used for heart rate estimation, was adapted and used to determine the optimal alignment between each peak in the respiratory signal and the template calculated by averaging the nearby peaks over a time window. Several modifications were needed to make it suitable for respiratory signals. Unlike PPGi signals, the morphology of the respiratory signal varies greatly according to the subject's breathing patterns. Preterm infants are known to have spontaneous breathing patterns[21]. Some infants, for example, may have a short inspiration phase followed by a prolonged expiratory phase; others may have a period of hold expiration followed by multiple expiratory flow peaks. The amplitude of each breath in the respiratory signal mainly depends on the depth of breathing or the volume of air inspired into the lungs. Hence, the criteria that were originally used for constructing a peak template from PPGi signals were too strict and not appropriate for respiratory signals.

Multi-scale dynamic time warping was carried out using a 15-second moving window w with a step size of 5 seconds. In order to measure the signal quality of each breath in the window, an average breath template was constructed. Once the template was calculated, the DTW distance was computed for each breath in the time window w. Let DTW be the distance between each breath and the window template, the SQI_{dtw} was defined as:

$$SQI_{\mathrm{dtw}}(k) = \begin{cases} 1 - DTW(k)/10 & \text{if } DTW(k) \leq 10 \\ 0 & \text{otherwise} \end{cases} \tag{11}$$

When the window template could not be calculated, the SQI_{dtw} of all breaths in the window w was set to 0 (poor quality). This modification was needed as variations in respiratory rate were much greater than variations in heart rate.

Once all the signal quality measures were calculated, a combined signal quality index (SQI_{breath}) was computed for the kth breath as:

$$SQI_{\mathrm{breath}}(k) = SQI_{\mathrm{act}}(k) \cdot SQI_{\mathrm{freq}}(k) \cdot SQI_{\mathrm{peak}}(k) \cdot SQI_{\mathrm{dtw}}(k). \tag{12}$$

SQI_{breath} was taken to be 0 (poor quality) during the periods of high subject motion ($SQI_{\mathrm{act}} = 0$) and abnormal instantaneous respiratory rate ($SQI_{\mathrm{freq}} = 0$). During a quiet and stable period, SQI_{breath} relied mainly on SQI_{dtw}.

References

1. Jeanne, V., De Bruijn, F. J., Vlutters, R., Cennini, G. & Chestakov, D. Processing images of at least one living being (2013). US Patent 8,542,877.
2. Kual-Zheng, L., Hung, P.-C. & Tsai, L.-W. Method and system for contact-free heart rate measurement (2013). US Patent App. 13/563,394.
3. Jones, M. J. & Rehg, J. M. Statistical color models with application to skin detection. *Int. J. Comput. Vis.* **46**, 81–96 (2002).
4. Breiman, L. Random forests. *Mach. learning* **45**, 5–32 (2001).
5. Bishop, C. M. *Pattern recognition and machine learning* (springer, 2006).
6. Kakumanu, P., Makrogiannis, S. & Bourbakis, N. A survey of skin-color modeling and detection methods. *Pattern Recognit.* **40**, 1106–22 (2007).
7. Bianco, S. & Schettini, R. Two new von Kries based chromatic adaptation transforms found by numerical optimization. *Color. Res. Appl.* **35**, 184–192 (2010).
8. Bianco, S. & Schettini, R. Computational color constancy. In *3rd European Workshop on Visual Information Processing*, 1–7 (2011).
9. Simonyan, K. & Zisserman, A. Two-stream convolutional networks for action recognition in videos. In *Advances in neural information processing systems*, 568–576 (2014).
10. Chatfield, K., Simonyan, K., Vedaldi, A. & Zisserman, A. Return of the devil in the details: Delving deep into convolutional nets. *arXiv preprint arXiv:1405.3531* (2014).
11. Villarroel, M. *Non-contact vital sign monitoring in the clinic*. DPhil thesis, University of Oxford (2017).
12. Villarroel, M., Jorge, J., Pugh, C. & Tarassenko, L. Non-contact vital sign monitoring in the clinic. In *2017 12th IEEE International Conference on Automatic Face & Gesture Recognition (FG 2017)*, 278– 285 (Washington, DC, 2017).
13. Chaichulee, S. *Non-contact vital sign monitoring in pre-term infants*. DPhil thesis, University of Oxford (2018).
14. ORuanaidh, J. J. K. & Fitzgerald, W. J. Retrospective changepoint detection. In *Numerical Bayesian Methods Applied to Signal Processing*, 96–121 (Springer, 1996).
15. Riemer, T., Weiss, M. & Losh, M. Discrete clipping detection by use of a signal matched exponentially weighted differentiator. In *IEEE Proceedings on SouthEastCon*, 245–248 (1990).
16. Müller, M. *Information retrieval for music and motion*, vol. 2 (Springer, 2007).
17. Fitriani. *Multiscale Dynamic Time and Space Warping*. Phd thesis, Massachusetts Institute of Technology (2008).
18. Salvador, S. & Chan, P. Toward accurate dynamic time warping in linear time and space. *Intell. Data Analysis* **11**, 561–580 (2007).
19. Jorge, J. *Non contact monitoring of respiration in the neonatal intensive care unit*. DPhil thesis, University of Oxford (2018).
20. Li, Q., Mark, R. G. & Clifford, G. D. Robust heart rate estimation from multiple asynchronous noisy sources using signal quality indices and a Kalman filter. *Physiol. Meas.* **29**, 15–32 (2008).
21. Te Pas, A. B. *et al.* Breathing patterns in preterm and term infants immediately after birth. *Pediatr. Res.* **65**, 352–356 (2009).
